# Simulation and experimentation of iron-doped liquid metal-based gallium oxide photocatalysts for environmental applications harnessing solar energy

**DOI:** 10.1007/s11356-025-36436-x

**Published:** 2025-05-08

**Authors:** Sayra Orozco, Espiridión Martínez-Aguilar, Carolina Belver, Jorge Bedia, Michel Rivero

**Affiliations:** 1 Posgrado de Ingeniería Química, Universidad Michoacana de San Nicolás de Hidalgo, Edif. V1, Ciudad Universitaria, Morelia, 58190 Michoacan Mexico; 2 Instituto de Investigaciones en Materiales, Unidad Morelia, Universidad Nacional Autónoma de México, Antigua Carretera a Pátzcuaro No. 8701, Morelia, 58190 Michoacan Mexico; 3 Chemical Engineering Department, Universidad Autónoma de Madrid, Campus Cantoblanco, Madrid, 58049 Spain

**Keywords:** Gallium oxides, Fe doping, Photocatalysis, DFT, Emerging pollutants

## Abstract

Gallium-based liquid metals (GLM) have emerged as promising materials for cutting-edge technologies. However, their increased use raises environmental concerns. Sustainable strategies, such as using them as nanophotocatalyst precursors, can help mitigate these impacts. In this work, gallium oxides doped with different atomic ratios of Ga:Fe (100:0, 80:20, 70:30, and 50:50) were synthesized from GLM, characterized, and evaluated in the degradation of an emergent pollutant (acetaminophen). The study considers theoretical modeling through the density functional theory. The photocatalysts were characterized by different techniques to investigate and corroborate the effect of iron on the structural, optical, and morphological properties. The results showed that Fe content influences the properties of gallium oxides. After Fe doping, the band gap of FeGO_x_ decreases to 3.21–2.78 eV. All materials showed photocatalytic activity in the visible region ($k_1 = 0.00324-0.00562$ min$^{-1}$ under visible illumination), reaching 65–80% mineralization under visible light, with similar performances under UVA light, making them suitable for use under solar radiation. Among the synthesized materials, FeGO_30_ displayed the best structural, optical, and morphological properties. Theoretical and experimental results are consistent. Several experiments were conducted using electron, proton, superoxide, and hydroxyl radical scavengers, suggesting that the reaction mechanism of Ac degradation could occur via HO^•^ radicals or oxidation through holes. Additionally, a band diagram is proposed for the FeGO_x_ materials.

**Supplementary Information:**

The online version contains supplementary material available at 10.1007/s11356-025-36436-x.

## Introduction

In recent years, gallium oxides have attracted attention due to their electrical, thermal, optical, and catalytic properties (Zhang et al. 2024; Mandal et al. 2025) and their importance in several applications. In electronics, gallium oxides are used in UV photodetectors (Wang et al. 2023a; Chen et al. 2025), high-power transistors (Chen et al. 2025), or Schottky diodes (Polyakov et al. 2018; Bhandari et al. 2019; Polyakov et al. 2019; Ku et al. 2024). Gallium oxides are also applied in environmental and energy sectors, as in methane conversion for production of ethane and hydrogen (Amano et al. 2024), in oxidation to CO_2_ (Tran et al. 2025; Liu et al. 2025), water splitting (Shen et al. 2025; Ammar Hassan Shah et al. 2025), and catalysis and photocatalysis (Orozco et al. 2023). Photocatalysis is a promising method for environmental remediation and energy conversion applications because it can degrade organic pollutants using an unlimited source: solar energy. In this field, gallium oxides have demonstrated photocatalytic activity for degrading dyes (Michel and Martínez-Preciado 2022b), emergent pollutants (Murguía et al. 2025), and halogenated organic compounds (Rajangam et al. 2021; Wu et al. 2024), among others, which are generally found in wastewater streams.

Wastewater pollution with emerging pollutants demands strategies to mitigate their environmental impact on different dimensions. Acetaminophen (N-(4-hydroxyphenyl ethanamide, C_8_H_9_NO_2_), a widely used pharmaceutical compound (Kim et al. 2025), emerges as a pervasive contaminant in wastewater streams since it is an antipyretic and analgesic drug for pain relief (Saidulu et al. 2025) and fever reduction. Indeed, it is one of the most often detected pharmaceutical products in sewage treatment plant effluents, surface water, and drinking water. Detection of this substance is more common in densely populated urban areas where drug use is expected to be higher and under steady growth (Ezugwu et al. 2023; Aguilar-Aguilar et al. 2023). In Mexico, acetaminophen has been found in surface water (9 456 ng L^–1^) and wastewater (67.2 ng L^–1^) (Vázquez-Tapia et al. 2022). Photocatalysis has been used as a solution to address this problem. The photocatalytic degradation of acetaminophen has been carried out using different photocatalysts (Wang et al. 2023b). Some of these investigations employed 2D TiO_2_ modified with co-doped (P, S)-g-C_3_N_4_ (P-S/co-doped g-C_3_N_4_/2D TiO_2_) (Cako et al. 2023), Titania fibers (FTiO_2_-350 and FTiO_2_-500) (García-Rollán et al. 2025), UiO-66-NH_2_ frameworks by N-cycloalkyl (UiO-66-NH-C_5_ and UiO-66-NH-C_6_) (Gómez-Avilés et al. 2023), 2D/0D Z-scheme Bi_2_O_3_/MnO_2_ (BMO) (2D/0D Bi_2_O_3_/MnO_2_) (Parida et al. 2023), TiO_2_-orange peel-derived biochar composite (TOBC20) (Mohtaram et al. 2024), BiOI-ZIF-8 nanocomposite (BiOI-ZIF-8) (Mengting et al. 2023), FeCo metal-organic framework (Pattappan et al. 2023), faceted TiO_2_ (Dudziak et al. 2023), Co_3_O_4_/TiO_2_ (Li et al. 2023), and Fe(III) doped TiO_2_ hollow submicrospheres (Fe/TiO_2_) (Huang et al. 2023). Gallium oxides have demonstrated effectiveness in the photocatalytic degradation of acetaminophen.

Ga_2_O_3_ has six polymorphs: $\alpha$, $\beta$, $\kappa$, $\delta$, $\epsilon$, and $\gamma$, being the $\beta$-Ga_2_O_3_ phase the most stable (Mandal et al. 2025). Gallium oxide is a wide bandgap semiconductor material (4.6–4.9 eV Mandal et al. 2025) compared to the TiO_2_ catalysts (3.2 eV) (Choi and Son 2017). Doping gallium oxides with Ag (Kitajima et al. 2021), Fe (Hany et al. 2019), Cu (Murguía et al. 2025), Mg (Kang et al. 2022), Cr, V, Mn (Li et al. 2022; Liu et al. 2023; Afandi et al. 2023), Nb (Yang et al. 2022), Ta (Wang et al. 2022), and Mo (Wang et al. 2023a) influences its conductivity and electrical, magnetic and optical performance by modifying the microscopic crystalline structure (Zeng et al. 2023).

Fe-doped gallium oxides are of special interest because iron owns the same tri-valence states with comparable ionic radii as the host Ga$^{3+}$ (Zeng et al. 2023). However, the electronic configurations of these two cations cause the properties to strongly depend on the mixed oxides of iron and gallium (Zhang et al. 2021; Zeng et al. 2023). In literature, it has been reported $\beta$-Ga_2_O_3_ doped with Fe (with concentrations lower than 5%) to explain the interaction between Fe and Ga atoms. Zeng et al. (2023) investigated the defect formation energies, charge transitional levels, and optical properties of Fe-doped $\beta$-Ga_2_O_3_. Their results demonstrated that (i) Fe dopant favors the octahedrally coordinated Ga site; (ii) Fe impurities can more easily replace Ga sites under O-rich conditions; and (iii) the presence of Fe-Ga complexes increases significantly the optical absorption in the Vis-IR region. Zhang et al. (2021) found similar results, and Fe$^{3+}$ ions are preferentially located in the octahedral sites over tetrahedral in the monoclinic structure. In turn, Bhandari et al. (2019) studied the Fe defect levels and the potential influence of additional defect centers in Ga_2_O_3_ by photoinduced electron paramagnetic resonance spectroscopy. They showed that the first optically induced change in Fe$^{3+}$ occurs at 1.2 eV, which is significantly larger than the calculated defect levels for Fe. Hany et al. (2019) investigated the optical and electrical properties along the *b*-axis of Fe-doped $\beta$-Ga_2_O_3_ by low-temperature cathodoluminescence (CL) spectroscopy, optical absorption spectroscopy, and current–voltage (IV) measurements. These authors show an optical bandgap of 4.45 eV and an intense blue to ultraviolet (UV) band in the blue range, from the as-grown Fe-doped $\beta$-Ga_2_O_3_. A low Fe content is considered in these studies.

Orozco et al. (2022a) investigated the photocatalytic activity of Fe-doped Ga_2_O_3_ at 5% content of Fe to show that the photoactivity improved due to the increase in the formation rate of HO$^\cdot$ radicals and the reduction of the electron–hole recombination rate. Nevertheless, they did not observe structural changes in the gallium oxide matrices due to the Fe low concentration. This study aims to explore the effects of higher Fe concentrations on gallium oxide photocatalysts for two main reasons. First, an absence of studies investigating the impact of higher Fe doping concentrations on gallium oxide properties. Second, experimental validation of theoretical investigations based on density functional theory suggests that iron oxides are favored under specific conditions.

Most photocatalysts have been synthesized using gallium salts as precursors. However, gallium liquid metal (GLM) has recently gained interest due to its excellent heat and electrical conductivity and its catalytic, optical, and optoelectronic properties (Daeneke et al. 2018; Tang et al. 2021; Tran et al. 2025). GLM is relatively non-toxic and has low viscosity, allowing it to flow easily at room temperature. There is an uprising trend in the development of flexible electronics based on liquid metals (LM) (Dickey 2014; Daeneke et al. 2018; Allioux et al. 2022; Afrin et al. 2023; Kim et al. 2024; Ohta et al. 2024), highlighting their relevance across a wide range of applications. An example is the outstanding development of soft electronics and robotics, which offers significant advantages for flexible and wearable sensors used in biomedical applications such as health monitoring (Sun et al. 2020) and artificial skin (Ren et al. 2020), both of which are expanding markets. In parallel, researchers are increasingly aware of the environmental impact of these technologies, which has boosted the development of new materials and approaches for the fabrication and disposal of flexible electronics (Luo et al. 2023), since end-of-life recovery is beneficial for the sustainable development of these technologies (Gao et al. 2018). As demand for gallium-based liquid metal-enabled technologies rises, GLM emerges as a suitable candidate as a precursor of gallium oxides (Syed et al. 2017; Fu et al. 2024; Murguía et al. 2025). Thus, it represents a way to recover and recycle gallium from e-waste streams, focusing on environmental applications.

In this work, we explore the use of gallium oxides doped with Fe ions for the photocatalytic degradation of acetaminophen as the model pollutant. Three different theoretical atomic ratios of Ga:Fe doping are considered: (80:20), (70:30), and (50:50). GLM is used as the precursor of the gallium oxides. The study assesses the influence of Fe content on the unit cell size, particle size, and shape, electrochemical, thermal, infrared, and UV–Vis properties. First-principle calculations based on the density functional theory (DFT) complement this study.

## Methodology

### Materials

Gallium-based catalysts were synthesized from procured highly pure precursor materials, wasted gallium metal liquid, hydroxide ammonium (NH_4_OH, 26–30%), nitrate ferric (Fe(NO_3_)_3_, 99.9%), and acetaminophen (C_8_H_9_NO_2_, analytic grade). Nitric acid (HNO_3_, 60–66% conc.) and sodium hydroxide (NaOH, 98%) were used for pH adjustment. These chemicals were purchased from Merk as A.C.S reagent grade and used as received without further purification.

### Synthesis methods

New catalysts synthesizing methods are shifting from conventional methods towards sustainable practices based on the green chemistry principles (Osman et al. 2024a). Within this framework, waste gallium liquid metal and water are utilized as a source of gallium and as a solvent in our research, respectively. Methods such as sonication (Parveen et al. 2018; Takano et al. 2021) and ultrasonic transducer (Murguía et al. 2025) have been tested as a way to decrease the time and temperature range of heat treatment—thus energy consumption during synthesis—and to improve the properties of gallium oxides (Rutherford et al. 2024). Another alternative is the formation of gallium-based heterojunctions with other elements that have lower energy requirements, or look for novel green synthesis techniques (Osman et al. 2024b) that might also lead to cost reduction and production increase. Therefore, gallium liquid metal-based catalysts were synthesized by simple precipitation of a solution of Ga ions with NH_4_OH at pH 10, coupled with an ultrasonic transducer (Orozco et al. 2022a). The gallium ion solution was prepared under acidic conditions, and 1 g of the gallium liquid metal was slowly added to a HNO_3_ solution (at 10% vol.) at 50 $^{\circ }\text {C}$ by 24 h. The gallium-iron oxy-hydroxides are labeled as FeGaO(OH)_x_, which do not represent the chemical composition of the material, and *x* stands for the theoretical Fe content. For FeGaO(OH)_x_ (x$=20$, 30, and 50 Fe content), an aqueous solution of nitrate ferric (0.08, 0.14, and 0.26 M) in acidic conditions was prepared and was slowly added to a gallium ion solution (0.28 M) under constant stirring. Subsequently, the NH_4_OH solution (10% in vol.) was added dropwise and kept under constant stirring until a pH 10 was reached. The theoretical Ga:Fe atomic ratios were 80:20, 70:30, and 50:50. This suspension was subjected to an ultrasonic process, using an ultrasonic transducer (SONICS Vibra Cell 750), at ${450\,\mathrm{\text {W}}}$ for 30 min. For GaO(OH), no Fe ion solution was added. Synthesized GaO(OH) and FeGaO(OH)_x_ (*x* $=20$, 30 and 50) materials undergone a 24-h aging process. Then, they were dried in an oven at ${50\,\mathrm{{ ^{\circ }\text {C}}}}$ overnight. Finally, these materials were treated at ${950\,\mathrm{{ ^{\circ }\text {C}}}}$ for ${3\,\mathrm{\text {h}}}$ in a TERLAB muffle (25 to ${1100\,\mathrm{{ ^{\circ }\text {C}}}}$ $\pm {1\,\mathrm{{ ^{\circ }\text {C}}}}$) in an oxygen atmosphere. The obtained photocatalysts are labeled as GO, FeGO_20_, FeGO_30_, and FeGO_50_. As with previous materials, the labels do not represent the chemical composition of the material and the subindex indicates the theoretical Fe content.

### Characterization

GO and FeGO_x_ ($x=20$, 30, and 50% in the theoretical atomic ratio Fe:Ga) were characterized by X-ray diffraction (XRD), X-ray photoelectron spectroscopy (XPS), thermogravimetric analysis (TGA), Fourier transform infrared (ATR-FTIR) spectroscopy, UV–Vis spectroscopy mode diffused absorbance, scanning and transmission electron microscopy (SEM-TEM), and electrochemical impedance spectroscopy (EIS).

The structure of photocatalysts was characterized by an X-ray diffractometer with a Bruker D2-Phaser diffractometer using CuK$\alpha$ radiation at 30 kV and 10 mA. Difractograms were scanned at $2\theta$ angles from 10 to ${80\,\mathrm{ ^{\circ }}}$ with ${0.5\,\mathrm{\sec }}$ per step and increments of ${0.0101\,\mathrm{ ^{\circ }}}$.

X-ray photoelectron spectroscopy (XPS) analyses were carried out in an ultra-high vacuum (UHV) system scanning XPS microprobe PHI 5000 VersaProbe II, with an Al K$\alpha$ X-ray source ($h\nu =1486.6$ eV) monochromatic with 100 $\upmu$m beam diameter, and a Multi-Channel Detector (MCD) analyzer. The XPS spectra were obtained at 45 $^{\circ }$ to the normal surface, pressure $5\times 10^{-7}$ Pa, constant analyzing energy (CAE) $E_0=117.40$, 11.75 eV survey surface, and high-resolution narrow scan. The surface samples were etched for 1 min with 0.5 kV Ar^+^ at 500 nA/mm$^{2}$. The peak positions were referenced to the background Ag $3d_{5/2}$ photopeak at 368.20 eV, with an FWHM of 0.81 eV, and C 1*s* hydrocarbon groups at 285.00 eV, Au $4f_{7/2}$ at 84.00 eV central peak core level position. The XPS spectrum was fitted with the program MultiPak PHI software and Spectral Data Processor, SDP v 4.1.

Thermogravimetric analysis was obtained using a TA Instruments Thermogravimetric Analyzer TGA 5500 under a nitrogen atmosphere (flow rate $={25\,\mathrm{\text {m}\text {L}\text {min}^{-1}}}$). The materials ($1.5-{3} \text {mg}$) were heated from 25 to 950 $^{\circ }\text {C}$ at a rate of 10 $^{\circ }\text {C}$min$^{-1}$.

The functional groups on the catalysts were obtained by ATR-FTIR spectroscopy. ATR-FTIR spectra were collected using a Thermo Scientific Nicolet iS10 FTIR spectrometer fitted with a Thermo Scientific Smart iTR ATR accessory with a diamond crystal and the OMNIC software. Powder samples were added directly onto the crystal for analysis at room temperature without applying pressure. Sixteen spectra were obtained and coadded for each sample covering a range of $4000-{650\,\mathrm{\text {c}^{-1}\text {m}}}$ at a spectral resolution of 4 c$^{-1}$m. A background spectrum was obtained by collecting a similar number of scans after cleaning the diamond crystal with acetone.

The morphology of the catalysts was analyzed using scanning electron microscopy (SEM) with a JEOL JSM IT300 microscope. Elemental mapping and EDX data were analyzed using AZtec analysis software. The morphological characteristics were studied also using a transmission electron microscope (TEM) JEOL JEM-ARM200F. TEM images were recorded using a FEI Talos F200X microscope at an accelerating voltage of 200 kV. The mean particle size was determined from a hundred different particles with the ImageJ software.

UV–Vis diffused absorbance spectra of the materials were measured with a Japan Shimadzu Spectrophotometer (2501 PC) UV–Vis in the wavelength $200-$800 nm range. A BaSO_4_ disk was used as a reference.

Electrochemical impedance spectroscopy (EIS) was carried out by scanning the frequency range from $10^{5}$ to $10^{-1}$ Hz at a fixed potential of $-1.2$ V. The semiconductor flat band potential was estimated from Mott-Schottky plots (Peñas-Garzón et al. 2023) using a three-electrode DropSens system in a 0.1 M Na_2_SO_4_ electrolytic solution (pH $\approx 6.2$ at 25 $^{\circ }\text {C}$) in a Potentiostat/Galvanostat (Metrohm Autolab model PGSTAT204). A fixed frequency of 100 Hz, an amplitude of 10 mV, and a potential range from $-1.5$ to 0.4 mV were used. Mott-Schottky (Eq. 1) was followed to calculate the semiconductor flat band potential, $V_{fb}$:

$$\begin{aligned} \frac{1}{C^2}=\frac{2}{\epsilon \cdot \epsilon _{0} \cdot e \cdot N_{D}}\cdot \left( V - V_{fb} -\frac{k T}{e}\right) \, . \end{aligned}$$

If $C^{-2} = 0$, it follows that

1 $$\begin{aligned} V_{fb} = V-\frac{k \, T}{e} \, , \end{aligned}$$

with *C* the capacitance at target voltage *V*; $\epsilon$ the semiconductor permittivity and $\epsilon _{0}$ the void permittivity; *e* the electron charge ($1.602 \times 10^{-19}$ J); *k* the Boltzmann’s constant ($8.617\times 10^{-5}$ eV K$^{-1}$); and *T* the temperature (298 K). $V_{fb}$ corresponds to the interception point of the tangent line with the abscissa axis ($1/C^{2}=0$). The conduction band potential ($V_{CB}$) is calculated with respect to NHE following the Nernst equation at pH 7 (Eq. 2).

2 $$\begin{aligned} V_{CB}= &  V_{fb \left( \text {Ag/AgCl},pH \right) } + \Delta V_{\left( \text {Ag/AgCl},\text {NHE} \right) }\nonumber \\ &  -0.059 \cdot \left( 7 - pH \right) \, , \end{aligned}$$

where $\Delta V_{ \left( \text {Ag/AgCl}, \text {NHE} \right) }$ represents the Ag/AgCl electrode potential with respect to NHE (0.21 V). Eventually, Eq. 3 allowed us to estimate the valence band potential ($V_{VB}$):

3 $$\begin{aligned} V_{VB}=V_{CB}+\frac{E_g}{e} \, , \end{aligned}$$

where $E_{g}$ is the semiconductor bandgap. Transient photocurrent analyses were carried out using an applied potential of 1 V and registering the transient current sampled every 0.1 s. A 60 W LED UV lamp with a $\lambda$ range between 385 and 400 nm (Onforu) was used as a light source with intervals of 15 s of on/off irradiation cycles. Linear sweep voltammetry (LSV) in the range of $-0.2$ to 1.2 V was preliminary performed to determine the adequate applied potential for transient photocurrent analyses.

### Analytic methods

The acetaminophen (Ac) degradation was evaluated from the intensity of the characteristic absorption peak at $\lambda _{char}= {0.243\,\mathrm{\mu \text {m}}}$. Absorption spectra of the samples in the wavelength range 0.2 to ${0.7\,\mathrm{\mu \text {m}}}$ were measured with Hach DR6000 Spectrophotometer, using a spectral interval of ${0.001\,\mathrm{\mu \text {m}}}$. The *pH* measurements were conducted with a HANNA *pH* meter, calibrated with standard buffers solutions of 4.01 and $7.01\pm 0.02$ (${25\,\mathrm{{ ^{\circ }\text {C}}}}$). The Ac mineralization degree was determined by total organic carbon (TOC) measured with the Hach (DR6000) direct method for low-range TOC Testing Tube Reactor/Curvette Tubes ($0.3-{20}\,\text {mg}\,\text {L}^{-1}$). The Fe(II) concentration, leached from the FeGO_x_, was measured by the *o*-phenanthroline colorimetric method after the reduction of Fe(III) with hydroxylamine chloride and by using a molar extinction coefficient of $1.11 \times 10^4$ M$^{-1}$ cm$^{-1}$ at 510 nm for the Fe(II)-phenanthroline complex.

### Experimental test

The photocatalytic activity of GO, FeGO_20_, FeGO_30_, and FeGO_50_ materials was studied by Ac degradation. The photolytic effect of the lighting source on acetaminophen degradation (without photocatalyst) was also considered. Experiments were carried out in a photoreactor with hydrodynamic batch operation and constant stirring to maintain the catalyst in suspension. Two low-power commercial lamps (13 W) were tested to illuminate the reaction space: *i*) a lamp emitting mostly at the visible region (400 to 700 nm) with several emission peaks, very low UV (3%) emission and a total radiative output of 2.87 W, and *ii)* a lamp emitting in the UVA region (320 to 400 nm), with a peak at 366 nm and a total radiative output of 2.25 W.

The effectiveness of heterogeneous photocatalysis depends on various factors, including catalyst and pollutant concentrations, the presence of an oxidizing agent, and pH, among others. Therefore, it is critical to study the impact of these parameters on the photocatalytic process. The experimental development involved two stages. In the first stage, numerous experiments were conducted to establish the optimal experimental conditions. These experiments were performed using GO and varying the catalyst concentration (0.50, 0.75 and 1.00 g L^–1^) and pH (3, 5 and 7). After defining the experimental conditions, we investigated the influence of Fe ion presence on the GO catalyst matrix, pH, and light source on the Ac degradation process. In both stages, the experimental procedure is as follows. The synthetic solution was prepared by dissolving 4.8 mg of Ac in 0.4 L of deionized water. The initial *pH* of the solution was adjusted to 3.00, 5.00, or 7.00 through a nitric acid solution (5% vol.) or sodium hydroxide (5% weight). At this time ($t=t_0$), the first sample was taken. The amount of GO or FeGO_x_, at an initial concentration of 0.50, 0.75 or 1.00 g L^–1^, was added into the solution. The reacting mixture was kept in agitation under dark conditions for 30 min to homogenize it and reach the adsorption equilibrium. At this time, a second sample was taken, the lamp was turned on, and the photocatalytic reaction started. The solution stayed under constant stirring for the whole duration of the photocatalytic process, and samples were taken at 15, 30, 60, 120, 180, 240, and 300 min. For the analysis of the Ac pollutant samples, catalyst particles were separated by centrifugation at 7500 rpm by 15 min. The photolysis of Ac was evaluated at pH 5 and 12 mg L^–1^ of Ac, in the absence of a photocatalyst under UVA and visible illumination conditions.

The Langmuir-Hinshelwood model is commonly used to evaluate the formation and/or disappearance rate of chemical species. This model involves the processes of external and intraparticle diffusion and surface reaction. In many cases, as in this work, the necessary information is incomplete, resulting in the use of pseudo-kinetic models to estimate the rates of formation or degradation of specific chemical species. Therefore, to evaluate the Ac degradation rate, it is assumed that (i) the photocatalyst concentration remains constant with respect to the Ac concentration, and (ii) the intermediate compounds formed during the process are generated and degraded during the reaction. This implies that the intermediates do not significantly affect the reaction. Under these assumptions, commonly found in literature, the Ac degradation rate depends only on the elapsed time or it has a linear dependence on the Ac concentration.

Experimental results were fitted to pseudo-zero- and first-order models. The equation used to describe the variation of Ac concentration ($C_{Ac}$) as a function of time for a pseudo-zero-order model is given by Eq. 4 as

4 $$\begin{aligned} -\frac{dC_{Ac}}{dt}=k_0 \, , \end{aligned}$$

and for a pseudo-first-order model by Eq. 5

5 $$\begin{aligned} -\frac{dC_{Ac}}{dt}=k_1~C_{Ac} \, , \end{aligned}$$

where the pseudo kinetic constants $k_0$ and $k_1$ are determined experimentally. These equations can be solved by specifying the initial concentration of the Ac solution $C_{Ac,0}$ at the time $t_0$. Equations 6 and 7 give the solutions for the concentration of the irradiated Ac solution, namely

6 $$\begin{aligned} C_{Ac,0}-C_{Ac}=k_0 \left( t - t_0 \right) \, , \end{aligned}$$

7 $$\begin{aligned} \ln \left( \frac{C_{Ac,0}}{C_{Ac}} \right) = k_1 \left( t - t_0 \right) \, . \end{aligned}$$

Finally, to investigate the photodegradation mechanism, electron (AgNO$_{3}$ at $5\times 10^{-4}$ M), hole (EDTA-2Na at $5\times 10^{-4}$ M), O_2_^–•^ (1,4-benzoquinone at $5\times 10^{-4}$ M) and HO^•^ (CH$_4$O at 10% Vol.) scavengers were tested.

## Numerical results

### Computational method

The Quantum-Espresso computational package (Giannozzi et al. 2009) was used to carry out the first-principle calculations based on the density functional theory (DFT). As a complement, Norm-conserving pseudopotentials were built with the generalized gradient approximation (GGA) in the Perdew-Burke-Ernzerhof (PBE) framework, used for exchange-correlation purposes (Rappe et al. 1990; Ramer and Rappe 1999). The description of the pseudopotentials was done with 13 valence electrons for Ga ($3d^{10} 4s^2 4p^1$), 8 valence electrons for Fe ($3d^6 4s^2$), and 6 for O ($2s^2 2p^4$). A plane-wave energy cutoff of 90 Ry was used throughout all calculations. $3 \times 10 \times 3$ and $3 \times 10 \times 6$ *k*-point meshes were used; the first was used for a $1 \times 1 \times 2$ supercell and the second for the conventional cell. All atomic positions and lattice constants were optimized until the magnitude of the residual energy and the force acting on each atom was below E-5 Ry/atom and E-3 Ry/a.u., respectively. Hubbard approximation was used.

The optical properties were determined employing the complex dielectric function, $\varepsilon \left( \vec {q},\omega \right)$ dependent on frequency $\left( \omega \right)$ and wave vector $\left( \vec {q} \right)$, whose complex elements can be represented as a $3 \times 3$ tensor, Eq. 8 (Wolfram and Ellialtioglu 2006):

8 $$\begin{aligned} \varepsilon \left( \vec {q},\omega \right) _{\alpha \beta } = \varepsilon _r \left( \vec {q},\omega \right) _{\alpha \beta } + i \, \varepsilon _i \left( \vec {q},\omega \right) _{\alpha \beta } \, , \end{aligned}$$

with $\varepsilon _r$ the real part and $\varepsilon _i$ the imaginary part, Eq. 9.

9 $$\begin{aligned} \varepsilon _i \left( \vec {q},\omega \right)= &  \frac{8 \pi ^2}{N \left( 2 a \right) ^3} \left( \frac{e}{m \omega } \right) ^2 \sum _{if} \left| \left\langle f \left| e^{i \vec {q} \cdot \vec {r}} \vec {a}_0 \cdot \vec {p} \right| i \right\rangle \right| ^2 \nonumber \\ &  \times \delta \left( E_f - E_i - \hbar \omega \right) f \left( E_i \right) \left[ 1 - f \left( E_f \right) \right] \, , \end{aligned}$$

where $\left| i \right\rangle$ is the initial state and $\left\langle f \right|$ the final state, $E_i$ and $E_f$ are the corresponding energies, *e* is the charge of the electron, and $\vec {p}$ is the dipole matrix. $f \left( E_i \right)$ is the probability that the initial state is occupied and $\left[ 1 - f \left( E_f \right) \right]$ is the probability that the final state is empty, $\alpha \beta = xy, \, xz, \text { or } yz$.

The real part $\varepsilon _r$ (Eq. 10) is determined by $\varepsilon _i$ through the Kramers-Kroning dispersion as follows (Wolfram and Ellialtioglu 2006):

10 $$\begin{aligned} \varepsilon _r \left( \vec {q},\omega \right) = 1 + \frac{2}{\pi } P \int _0^{\infty } \frac{\omega ^{'} \varepsilon _i \left( \vec {q},\omega \right) }{ \omega ^{' 2} - \omega ^2 } d \omega ^{'} \, , \end{aligned}$$

with *P* as the main value.

### Structure

The conventional cell of $$\beta $$–Ga_2_O_3_ belongs to the space group *C*2/*m* (#12), with 4 unit formulas of Ga_2_O_3_ in the unit cell (Geller 2004). Under this space group, Ga is found in two crystallographically independent Wyckoff positions, 4i (0,0,0) and 4i $$ \left( 1/2, 1/2, 0 \right) \pm \left( x,0,z \right) $$. In the first site, Ga^3+^ is in coordination 4 (GaO_4_), and in the second, in coordination 6 (GaO_6_); these positions are labeled as Ga1 and Ga2, respectively. On the other hand, there are three inequivalent positions for O_2_^–^, labeled OI, OII, and OII in Fig. 2. The lattice parameters are $$ a = 12.214$$ Å, $$b = 3.037$$ Å  and $$c = 5.798$$ Å , with $$ \alpha = \gamma = {90\,\mathrm{ ^{\circ }}}$$ and $$ \beta = {103.83\,\mathrm{ ^{\circ }}} $$.

The possible Ga occupation sites were considered when doping with Fe$^{3+}$ ions, that is two occupation sites such as Fe1 and Fe2. Using the special quasirandom structures (SQS) model (Okhotnikov et al. 2016) allows to build structures that describe the possible occupation sites of the Fe$^{3+}$ ion within the $\beta$–Ga_2_O_3_ matrix. For 12.5%, a $1 \times 1 \times 2$ cell was built, while for 25% and 50% in Fe concentration, the $\beta$–Ga_2_O_3_ unit cell was used. The number of combinations that originate from the possible occupation sites for the Fe_4_Ga_4_O_12_ system $\left( 50\% \right)$ is 36, which results in only 12 possible structures after accounting symmetries. In the case of 25%, Fe_2_Ga_6_O_12_, 16 possible combinations arise, with symmetry reducing to only four configurations. Finally, for 12.5%, Fe_2_Ga_14_O_24_, there are 64 possible configurations, from which only eight possible structures exist by symmetry reduction.

**Fig. 1:**
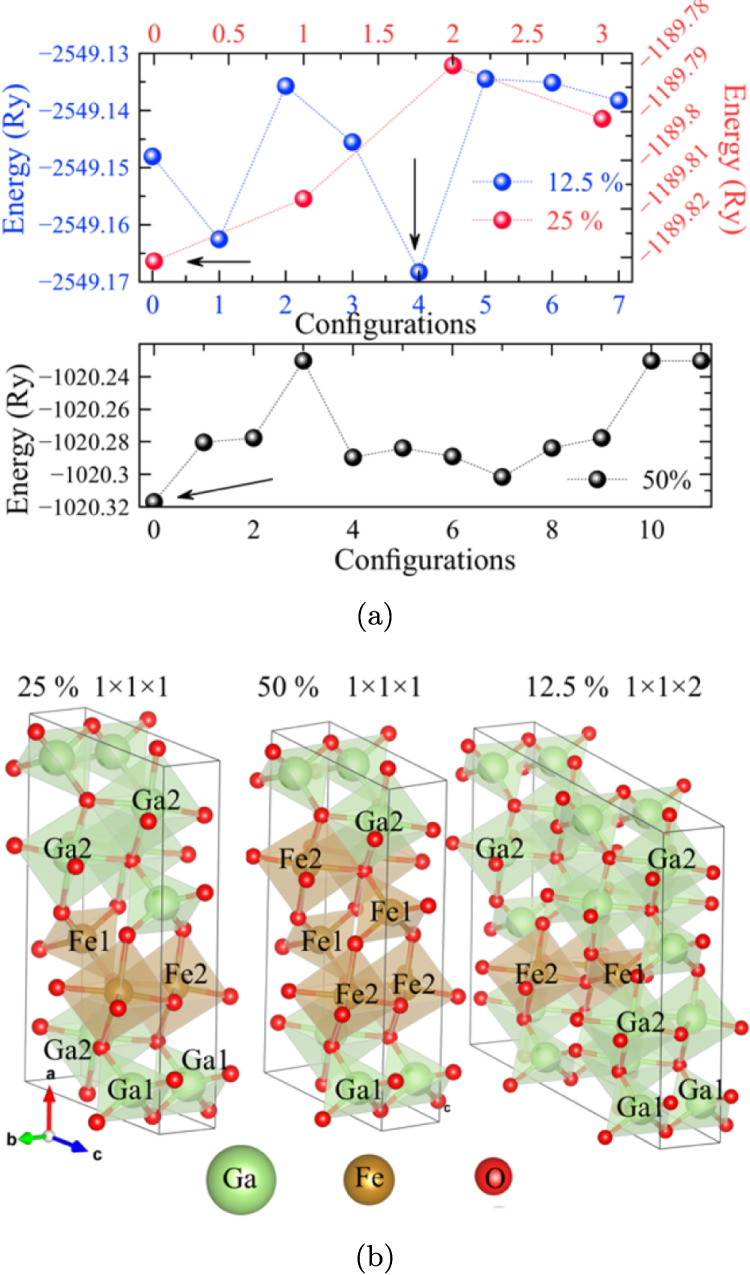
**a** Energy vs. configuration for the different Fe concentrations. **b** Completely relaxed minimum energy configurations for 25, 50, and 12.5% Fe concentration. The arrows in **a** indicate the minimum energy configurations. In **b**, the ion inside the tetrahedron represents Ga1, while the ions in the center of the octahedron represent Ga2; this is identical for Fe ions

In Fig. 1, the energy is shown compared against the possible configurations. The minimum energy values were used as the criteria for selecting the structure to study. Figure 1b shows the resulting structure for each case.

Given the addition of a magnetic ion such as Fe, we considered the possible states non-magnetic (NM), antiferromagnetic (AFM), and ferromagnetic (FM), for the most stable configurations. As in the previous analysis, energy is used as a comparison parameter. Figure S1 (see supplemental material) identifies the energetically favorable structures. First, the 12.5% Fe structure is stabilized as an AFM system. When incorporating 25% Fe, a marked FM behavior is distinguished, and finally, with a 50% Fe content, the system behaves like AFM. It should be noted that the energy difference between the FM and AFM states, both for the system with 12.5% and 50% Fe, are indicative of a ferrimagnetic state, which is out of the scope in this work, but opens the possibility for new magnetic investigations in the $\beta$–Ga_2_O_3_ doped with Fe.

The lattice parameters, completely relaxed, are shown in Table 1. It can be seen that the calculated values are overestimated by $\sim 1.8\%$ to the experimental values (Mohamed et al. 2011). This behavior is expected when using the generalized gradient approximation (Wang and Pickett 1983; Perdew and Levy 1983) and is in good agreement with the experimental and numerical (from first-principles calculations Ding et al. 2024) values. The comparison between the calculated parameters reveals an increase in the lattice parameters as the Fe concentration increases, as observed by Zhang et al. (2021). This occurs due to the difference in the ionic radius between Fe1 in its IV coordination (0.49) and Fe2 in its VI coordination (0.645), while Ga1 presents a smaller radius for its IV coordination (0.47). The same occurs for Ga2 in its VI coordination (0.62). Thus, the increase in the volume of Fe-doped systems is expected.

**Table 1:** Lattice parameter for the different Fe-doped $\beta$–Ga_2_O_3_ configurations (theoretical percentages) and the gap value

System	$a \,$ (Å)	$b \,$ (Å)	$c \,$ (Å)	$\beta$ $(^{\circ })$	$E_{g}$
$\beta$–Ga_2_O_3_ Exp (Rappe et al. 1990)	12.214	3.037	5.798	103.83	4.448
$\beta$–Ga_2_O_3_ Num (Ding et al. 2024)	12.109	3.002	5.745	103.97	4.92
$\beta$–Ga_2_O_3_	12.439	3.091	5.876	103.83	4.704
12.5%Fe: $\beta$–Ga_2_O_3_	12.474	3.102	5.921	103.91	4.096
25%Fe: $\beta$–Ga_2_O_3_	12.478	3.103	5.923	103.98	3.703
50%Fe: $\beta$–Ga_2_O_3_	12.485	3.104	5.927	103.83	2.822

For all cases, $\alpha = \gamma = {90\,\mathrm{ ^{\circ }}}$

**Fig. 2:**
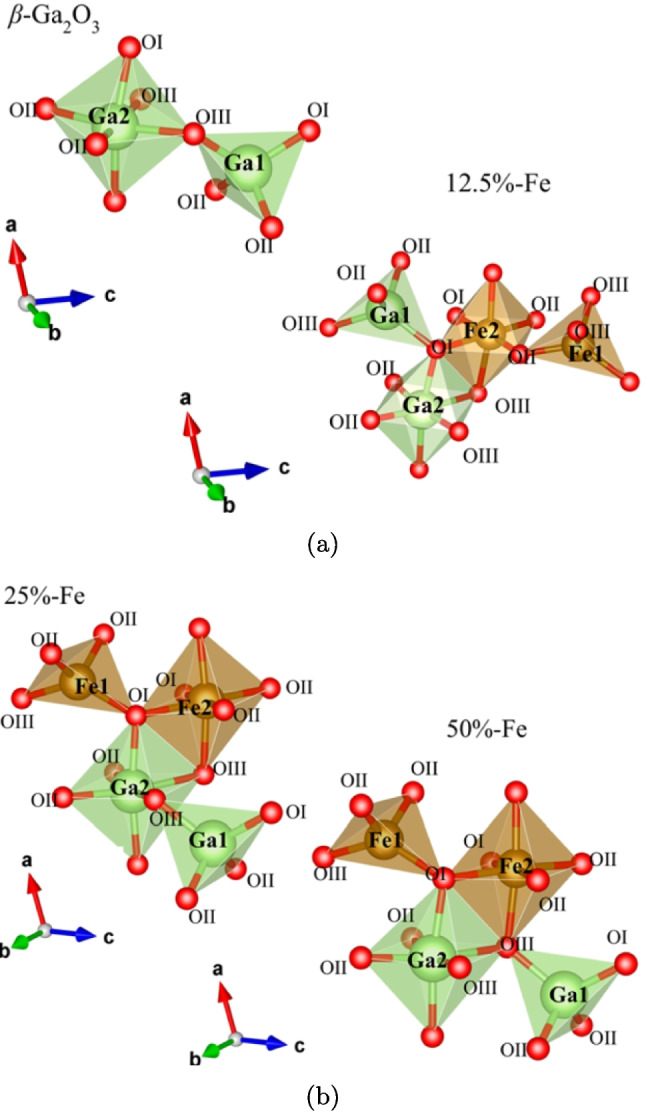
Interaction of the tetrahedral (Ga1, Fe1) and octahedral (Ga2, Fe2) sites with the closest oxygen for **a** $\beta$–Ga_2_O_3_ and 12.5%Fe: $\beta$–Ga_2_O_3_ and **b** 25%Fe: $\beta$–Ga_2_O_3_ and 50%Fe: $\beta$–Ga_2_O_3_. The labels OI, OII, and OIII indicate the three inequivalent oxygen sites in the *C*2/*m*1 space group

**Table 2:** Lattice parameter for the different Fe-doped $\beta$–Ga_2_O_3_ configurations, bandgap, and formation energy (F.E.)

System	Database	$\beta$–Ga_2_O_3_	12.5% Fe: $\beta$–Ga_2_O_3_	25% Fe: $\beta$–Ga_2_O_3_	50%Fe: $\beta$–Ga_2_O_3_
Ga1-OI	1.852	1.868	1.951	1.930	1.889
Ga1-OII	1.833	1.870	1.914	1.919	1.873
Ga1-OIII	1.803	1.904	1.932	1.962	1.797
Ga2-OI	2.023	2.037	2.157	2.103	2.085
Ga2-OII	2.077	1.973	2.030	2.048	2.019
Ga2-OIII	1.978	2.100	2.032	2.153	2.103
Fe1-OI	—	—	1.889	1.823	1.904
Fe1-OII	—	—	1.807	1.919	1.906
Fe1-OIII	—	—	1.917	1.962	1.932
Fe2-OI	—	—	2.105	2.104	2.045
Fe2-OII	—	—	1.991	2.008	1.971
Fe3-OIII	—	—	2.050	2.066	2.068
F.E. (eV)		$-$3.99	$-$6.46	$-$9.99	$-$5.65

For all cases, $\alpha = \gamma = {90\,\mathrm{ ^{\circ }}}$

Besides, it was possible to distinguish variations in the bond length when doping with Fe. These values were taken between the occupation sites of Ga1, Ga2, Fe1, Fe2, and the closest neighboring oxygen; see Table 2 and Fig. 2. It was observed a reduction in the bond length in Fe-O compared to Ga-O bonds, which can be associated with the increase in covalence due to the difference in electronegativities, Ga (1.7), Fe (1.8), and by the increase in the charge density of the Fe ions. Furthermore, as part of structural stability, we analyzed the formation energy for the different structures under investigation. The expression given by Eq. 11 was used for this purpose:

11 $$\begin{aligned} E_f = E_{Fe:\beta -Ga_2O_3} - E_{\beta -Ga_2O_3} - n_{Fe}\mu _{Fe} + n_{Ga}\mu _{Ga} \, , \end{aligned}$$

where $E_f$ is the formation energy, $E_{\beta -Ga_2O_3}$ is the total energy of the pristine system, and $E_{Fe:\beta -Ga_2O_3}$ is the energy of the system doped with iron. $n_{Fe}$, $\mu _{Fe}$, $n_{Ga}$, and $\mu _{Ga}$ are the corresponding number of atoms and the chemical potential for iron and gallium, respectively. The chemical potentials for metallic Ga and Fe in their BCC structure were obtained from the theoretical model.

**Fig. 3:**
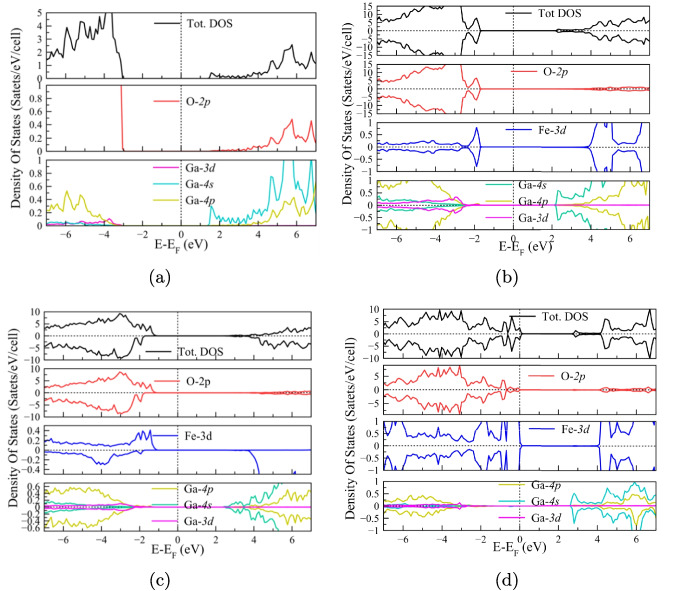
Density of total and projected states on the orbitals of **a** $\beta$–Ga_2_O_3_, and doped at **b** 12.5%, **c** 25%, and **d** 50%. The Fermi level is set to 0 for all cases. For sections b–d, the two high and low spin channels are shown

The formation energy (shown in Table 2) obtained for $\beta -$Ga$_2$O$_3$ is similar to the values obtained by other authors (Usseinov et al. 2021; He et al. 2006). In all cases, the formation energy is lower than that of the pristine system, suggesting that Fe incorporation is thermodynamically favorable. Doping with Fe at a concentration of 25% presents the most negative formation energy, suggesting a higher thermodynamic stability among the three doping concentrations. For 12.5 and 50%, the stability decreases, which indicates that the optimal doping lies between these values.

### Density of states

Figure 3a shows the density of states for pristine $\beta$–Ga_2_O_3_. Note that the O$-2p$ states are predominant in the valence band maximum (VBM), while the Ga$-4s$ states are in the majority in the conduction band minimum (CBM) so that the energy difference between these states causes an indirect band gap of 4.704 eV and a direct band gap of 4.711 eV (see Fig. S2 in SM). Under these equilibrium conditions, $\beta$-Ga$_2$O$_3$ exhibits a majority of Ga-4*s* states above the Fermi level by approximately 1.5 eV, which drives and promotes its *n*-type conductivity. In the case of 12.5% Fe doping, the incorporation of Fe$^{3+}$ ions produces a shift of the Ga states towards lower levels below the Fermi level, particularly below $-2$ eV. Since Ga, like Fe, donates electrons to oxygen, and since Ga has a closed $3d^{10}$ shell, while Fe has a $3d^5$ shell, the introduction of states below the Fermi level is allowed, between 0 and $-2.2$ eV, producing a hybridization between the O$-2p$ and Fe$-3d$ orbitals. The VBM is consecutively governed by the O$-2p$ states, and the Ga$-4s$ states are governed by the CBM (see Fig. 3b). Incorporating 12.5% Fe into $\beta$–Ga_2_O_3_ produces a *p*-type extrinsic semiconductor with a 4.096 eV gap. For higher theoretical Fe doping, 25% and 50% (Fig. 3c and d, respectively), a similar behavior is observed, but with an increase in the number of states/eV, that is, an increase in the charge density associated with the 3*d* Fe orbitals. The charge increases as the Fe concentration increases. In all cases, the VBM is governed by the O$-2p$ states, while the CBM is mainly governed by the Ga$-4s$ states. The *p*-type characteristic after doping with Fe is key in photocatalysis. As the holes have a high oxidizing potential, the degradation of contaminants is favored by the states introduced by the Fe$^{3+}$ ion (Chauhan et al. 2024), particularly the Fe-3*d* states. In addition, the presence of these states acts as electronic transition centers, reducing the bandwidth of the pristine system. Thus, the gap reduction for all Fe doping is associated with the Fe$-3d$ states that are introduced below the Fermi level, so charge transfer between these states is possible. As shown in Table 1, the gap value is attenuated with the increase in Fe concentration from 4.711 eV for the pristine $\beta$–Ga_2_O_3_, up to 2.822 eV for the maximum Fe concentration.

Due to the shift of the Ga states below the Fermi level, and the introduction of Fe states near the Fermi level, the availability of electronic states of Ga for the formation of Ga-O bonds is affected and is favored the formation of Fe-O bonds. Therefore, there is a high probability that such conditions influence the formation of Fe oxides, particularly for concentrations greater than 25% Fe.

The analysis of the Bader charge (Table S1 in the supplementary material) for $\beta -$Ga$_2$O$_3$ and the different levels of Fe doping have been used to evaluate the Fe oxidation state and its influence on the electronic structure. As observed in Table S1, the undoped system presents a “homogeneous” charge for Ga ions and oxygen. The difference between the ideal value of 3+ for Ga and $-$2 for oxygen allows us to distinguish that there is no purely ionic behavior. Instead, there is a mixture of ionic and covalent characters. With the incorporation of Fe at 12.5%, it is distinguished that the Fe ions present a charge of $\sim 2.9$ *e*, indicating that the system is stable without a significant addition of free carriers. In addition, the variation in the charge of the oxygen ion implies that this ion has gained less charge than expected for a purely ionic system, which is associated with bonds with a degree of covalence. A similar behavior is observed when doping with 50% of Fe. In contrast, with 25% doping, gallium, and oxygen partially reduce their charge to ranges between 1.86–1.95 *e* and $-$1.27 to $-$1.36 *e*, respectively. This implies that electron redistribution is consistent with the density of states. In addition, Fe ions have a charge between 2.5 and 2.6 *e*, indicating a mixed charge regime of Fe that enables the coexistence of Fe$^{2+}$ and Fe$^{3+}$. When Fe is reduced, the redistribution of charges affects the effective charge of Ga, favoring hybridization between Ga-O. Furthermore, the reduction of Fe and O indicates that oxygen does not extract as much charge from Fe as occurs for doping above and below 25%. The mixed charge character in Fe implies that fewer electrons are being extracted, which favors self-compensation, that is, the reduction of the charge in both Ga and O, leading to the formation of intrinsic defects associated with oxygen vacancies. These changes can favor optical absorption with bandgap reduction, as shown in the state density. On the other hand, the probable formation of oxygen vacancies could influence surface reactivity and favor phase segregation.

**Fig. 4:**
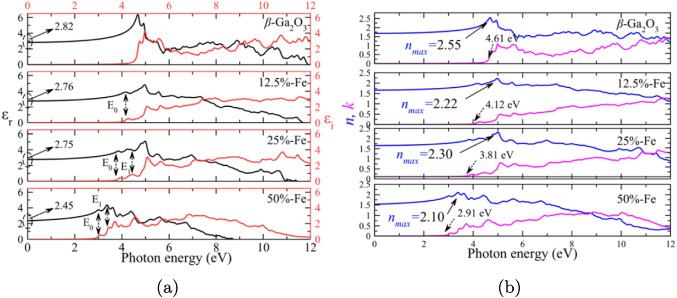
**a** Complex dielectric function of $\beta$–Ga_2_O_3_ and all doping, from 0 to $50\%$ Fe. **b** Refractive index and extinction coefficient for all configurations under study, from 0 to $50\%$ Fe doping

### Dielectric function

The optical properties of Fe-doped $\beta$–Ga_2_O_3_ were calculated from the dielectric function. The real part $\left( \varepsilon _r \right)$ provides information related to the ability of the material to store energy, while the imaginary part $\left( \varepsilon _i \right)$ helps to explain the electronic transitions from the occupied states of the valence band to the unoccupied states of the conduction band. From an optical point of view, the real and imaginary parts of the dielectric function are best related to each other by the refractive index $\left( n \right)$ and the extinction coefficient $\left( k \right)$, Eqs. 12 and 13, which can be described from the dielectric function as follows:

12 $$\begin{aligned} n= &  \left( \frac{\varepsilon _1}{2} + \frac{1}{2} \left[ \varepsilon _1^2 + \varepsilon _2^2 \right] ^{\frac{1}{2}} \right) ^{\frac{1}{2}} \, ,\end{aligned}$$

13 $$\begin{aligned} k= &  \left( - \frac{\varepsilon _1}{2} + \frac{1}{2} \left[ \varepsilon _1^2 - \varepsilon _2^2 \right] ^{\frac{1}{2}} \right) ^{\frac{1}{2}} \, . \end{aligned}$$

The decomposition of the dielectric function in the different crystallographic axes, real and imaginary parts, makes evident the existence of an optical anisotropy for all considered configurations so that the absorption depends on the polarization of the incident light; this is shown in Fig. S3 from the SM. On the other hand, Fig. 4a compares the real and imaginary parts of the dielectric function for all the cases under study. For pristine $\beta$–Ga_2_O_3_, the static dielectric constant $\left( \varepsilon _r \left( 0 \right) \right)$ is identified at 2.82, and this value is attenuated with the Fe concentration, up to 2.45 when the theoretical $50\%$ Fe concentration is reached. Furthermore, from $12.5\%$ Fe, small peaks below the absorption value of the pristine system can be distinguished, particularly in the imaginary part of the dielectric function. For the configuration with $12.5\%$ Fe, the first peak $\left( E_0 \right)$ is observed near 4 eV. This is coincident with a combination of Ga$-4s$ and Fe$-3d$ states in the CBM (see Fig. 3). In this case, the charge transfer occurs between the O$-2p$ states and the combination of Ga$-4s$ and Fe$-3d$ orbitals under the action of photons, accentuating the role of Fe as a gap-reducing agent in $\beta$–Ga_2_O_3_. The behavior is similar for doping with $25\%$ Fe, with $E_0$ just above the gap value (3.703 eV) and $E_1$ above 4 eV. The first peak indicates the charge transfer between the O$-2p$ from the VBM to the Ga$-4s$ states in the CBM. However, the $E_1$ peak is more pronounced and represents an absorption increase. This occurs by charge transfer between the O$-2p$ states and the combination of the Ga$-4s$ and Fe$-3d$ states, where the concentration of Fe$-3d$ orbitals is the majority for this range of energies. The same occurs for the maximum Fe concentration, with the difference that the charge transfer is associated with the combination of the O$-2p$ and Fe$-3d$ states in the VBM towards the Ga$-4s$ states in the CBM. Finally, the refractive index $\left( n \right)$ and the extinction coefficient $\left( k \right)$ are shown in Fig. 4b, where it is highlighted that the maximum value of *n* decreases as the Fe concentration increases. The coefficient of extinction shows the energy values where the material starts the photon absorption process. The process starts at 4.62 eV for the pristine configuration and decreases to 2.91 eV when the maximum theoretical Fe concentration is reached. Note that the reduction in the value of the peaks (for the dielectric function and in *n* and *k*) and the reduction in the photon absorption energy are products of the increase in states introduced by the concentration of Fe. This implies an increase in the concentration of charge carriers when Fe is introduced as a GaO dopant.

## Experimental results

### Characterization

The diffraction peaks in the XRD pattern of GaO(OH) can be indexed according to JCPDS 06-0180, which has a crystallized orthorhombic structure (Reddy et al. 2015; Huang et al. 2017; Sharma et al. 2020). Figure S4 (in the Supplementary Material) shows the XRD patterns for GaO(OH), FeGaO(OH)_20_, FeGaO(OH)_30_, and FeGaO(OH)_50_. For these materials, the representative peaks to crystalline planes are (110), (130), (111), and (240) at diffraction angles, $2\theta =21.50$, 33.78, 37.29, and $54.02 { ^{\circ }}$, respectively. In turn, the XRD pattern for GO photocatalysts shows the formation of $\beta$ phase GO at ${950\,\mathrm{{ ^{\circ }\text {C}}}}$. This sample has higher order diffraction lattice planes, implying a more crystalline material than can be readily indexed to the monoclinic structure of $\beta$-Ga_2_O_3_ phase according to JCPDS 76-0573 (see Fig. S5 in the SM) (Quan et al. 2010; Guo et al. 2017; Cheah et al. 2020; Sharma et al. 2020). The representative peaks corresponding to the crystalline planes are (401), (002), (111), and (512) at diffraction angles $2\theta =30.47$, 31.87, 35.43, and $64.74 { ^{\circ }}$, respectively.

**Fig. 5:**
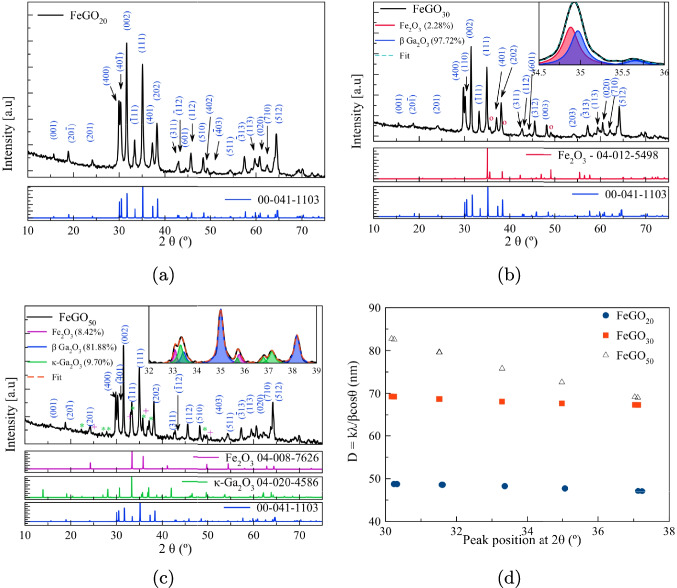
**a** X-ray diffraction pattern for the sample doped with 20% iron (FeGO_20_), **b** Ga$_2$O$_3$ doped with 30% Fe (FeGO_30_), **c** Ga$_2$O$_3$ doped with 50% Fe (FeGO_50_), and **d** crystallite size depending on the peak’s position at 2$\theta$

From the X-ray diffraction patterns from Fig. 5a–c, polycrystalline $\beta$-Ga$_2$O$_3$ phases (ICDD PDF 00-041-1103) were indexed with space group *C2/m* for all FeGO$_X$ compounds. No secondary phases are observed when doping with 20% Fe. However, for a 30% Fe doping, an orthorhombic phase of Fe$_2$O$_3$ is distinguished, with a phase amount of 2.28% (ICDD PDF 04-012-5498); the contribution is shown in the decomposition of the peaks between 34 and 36$^{\circ }$ in 2$\theta$. In comparison, the sample with 50% Fe presents multiple secondary phases, a phase of R-3c Fe$_2$O$_3$ with 8.42% (ICDD PDF04-008-7626) and 9.70% for $\kappa$-Ga$_2$O$_3$ (ICDD PDF-04-020-4586). As established in the numerical section, concentrations greater than 25% of Fe favor the formation of iron oxides and other GaO species. In Fig. 5c, the deconvolution is used to identify each peak’s contribution to the diffraction pattern. For all cases, the highest percentage corresponds to the $\beta$-Ga$_2$O$_3$ structure.

The lattice constants of the Fe_x_GO photocatalysts in Table 3 indicate an increasing trend in the lattice parameters with higher Fe concentration, consistent with first-principles calculations. The increase in the lattice constants is associated with the difference in ionic radii between Fe and Ga. However, small variations in the lattice parameter and angle $\beta$ are indicative of a faint structural distortion. On the other hand, the secondary phases indicate the formation of iron oxides as the concentration of Fe increases since the Fe electronic configuration favors the loss of electrons. The same is observed in the density of states, shown in Fig. 3, where the O-2*p* states are hybridized more easily with the Fe-3*d* states than with those of Ga.

**Table 3:** Lattice parameter for the different Fe-doped $\beta$–Ga_2_O_3_ photocatalysts synthetized by precipitation coupled by ultrasonic process. For all cases $\alpha = \gamma = {90\,\mathrm{ ^{\circ }}}$

System	$a \,$ (Å)	$b \,$ (Å)	$c \,$ (Å)	$\beta \, (^{\circ })$	$E_{g}$ Exp
Fe_20_GO	12.292	3.048	5.824	103.795	3.21
Fe_30_GO	12.323	3.054	5.838	103.759	3.18
Fe_50_GO	12.324	3.054	5.838	103.746	2.78

**Fig. 6:**
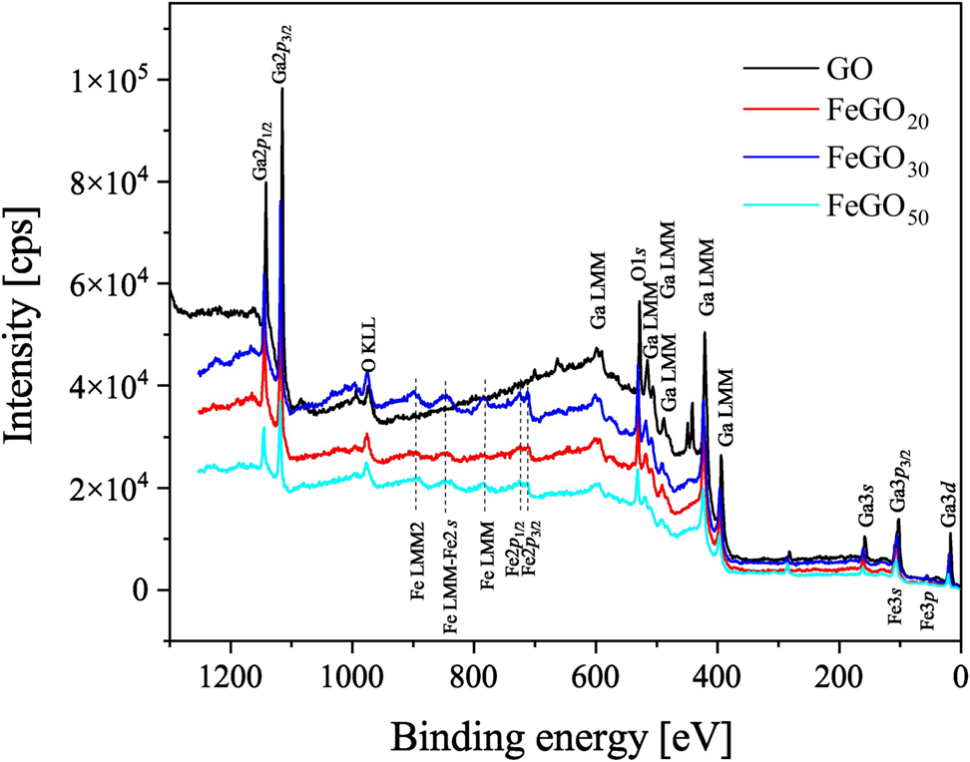
XPS spectra of gallium oxide (GO) and gallium oxide doped with Fe, FeGO_20_, FeGO_30_, and FeGO_50_ synthesized by alkaline precipitation assisted with ultrasonic transducer and treated at 950 $^{\circ }\text {C}$

The crystallite size was determined from the Scherrer equation, Eq. 14,

14 $$\begin{aligned} D=\frac{K\lambda }{\beta \cos \theta } \, , \end{aligned}$$

with *D* the crystallite size, *K* a constant taken as 0.89, $\lambda$ the wavelength, $\beta$ the width at half height of the diffraction peak, and $\theta$ the diffraction angle. Figure 5d shows the crystallite size depending on the peak in $2\theta$ for the three theoretical Fe concentrations. This figure shows a crystallite size between 50 and 80 nm and demonstrates that it increases with the concentration of Fe.

XPS spectra of gallium oxides (GO and Fe-doped gallium oxides (FeGO_20_, FeGO_30_ and FeGO_50_) photocatalysts after Ar^+^ ion cleaning are shown in Fig. 6 depicting similar spectra. For Fe-doped gallium oxides, the spectra evidenced photoelectron peaks corresponding only to Ga, O, and Fe, corroborating no trace elements contamination in the corresponding photocatalysts. A minor C component was observed in all cases due to a shorter Ar^+^ ion cleaning performed to reduce substoichiometric C species absorption (Delgado et al. 2022). The peaks at 18.2 eV for elemental Ga and at 20.6, 107.6, 161.6, 1118.6, and 1144.6 eV revealed the $+3$ oxidation state of gallium (3*d*, $3p_{3/2}$, 3*s*, $2p_{3/2}$, $2p_{1/2}$) (Parveen et al. 2018; Sudrajat and Nguyen 2020; Ramana et al. 2021). The spectra show a peak centered at 532 eV, corresponding to O1*s* (Orozco et al. 2023) and peaks situated at 710.2 and 723 eV corresponding to Fe2$p_{3/2}$ and Fe2$p_{1/2}$, respectively (Abdelmoula et al. 2023). The composition of GO, FeGO_20_, FeGO_30_, and FeGO_50_ was determined by XPS and is shown in Table 4. The average Ga:Fe ratio for FeGO_20_, FeGO_30_, and FeGO_50_ photocatalysts was 94.28 : 5.72, 87.47 : 12.53, and 79.60 : 20.40, respectively.

**Fig. 7:**
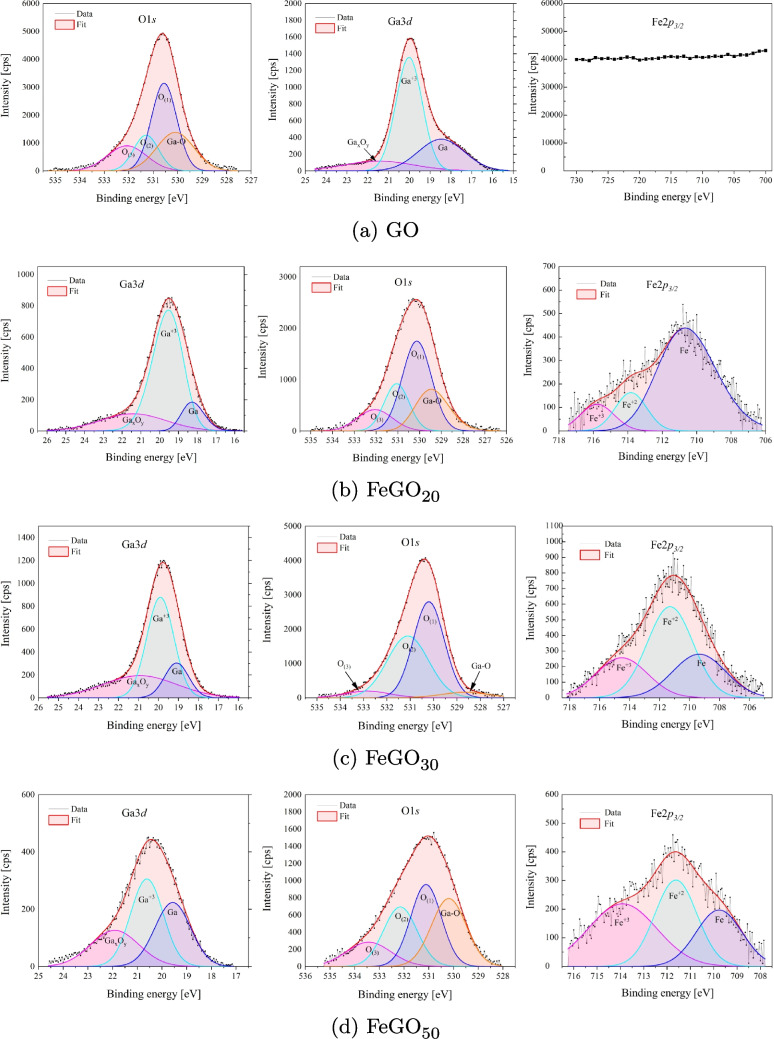
Deconvolution for GO and the different Fe-doped $\beta$-Ga_2_O_3_ photocatalysts

The deconvolution of Ga3*d*, O1*s*, and Fe2$p_{3/2}$ is presented in Fig. 7. For chemical analysis of these materials, the Ga3*d* region offers key information on the oxidation state of Ga. Ga3*d* peak was deconvoluted into three individual curves (see Fig. 7) for the groups listed in Tables 5 and 6, where its relative content in GO and FeGO is included. For gallium, it presents peaks assigned to elemental Ga, Ga$^{3+}$, and Ga$_x$O$_y$ (Qin et al. 2012; Zhang et al. 2021). In turn, the deconvolution of O1*s* shows oxygen associated with oxides and three-site oxygen constructions: Ga-O, O$_{(1)}$, O$_{(2)}$, O$_{(3)}$, that can be associated with different chemical states or chemical bonding environments around oxygen (Chikoidze et al. 2024).

**Table 4:** Atomic composition of GO, FeGO_20_, FeGO_30_, and FeGO_50_

Photocatalyst	Composition					
	XPS			EDS		
	Ga (%)	O (%)	Fe (%)	Ga (%)	O (%)	Fe (%)
GO	52.2	47.8	–	23.99	76.01	–
FeGO_20_	49.5	47.5	3.0	32.03	63.07	4.90
FeGO_30_	38.4	56.0	5.5	21.72	71.61	6.67
FeGO_50_	42.9	46.1	11.0	28.08	65.58	6.34

The deconvolution of Fe$2p_{3/2}$ peak reveals the Fe bond characteristics and the chemical state in Ga_2_O_3_ for the three different species of Fe:Fe, FeO, and Fe_2_O_3_, corroborating XRD results (see Fig. 5). Fe$2p_{3/2}$ peak present a symmetric shape, due to the presence of Fe ions in multiple chemical/oxidation states, as well as satellite peaks (see Fig. 6) (Ramana et al. 2021). The increase of Fe content in the Ga_2_O_3_ matrices changes the electronic structure, as reflected in the intensity of the Fe$2p_{3/2}$ peak components, indicating that Fe compounds, as oxides, give rise to higher energy bands. According to the results, the Ga_2_O_3_ photocatalysts doped with Fe exhibit mixed valence states (Fe$^{3+}$ and Fe$^{2+}$). In FeGO_20_ and FeGO_30_, materials predominate the Fe$^{2+}$ chemical state, with a lower proportion as Fe$^{3+}$, corroborating X-ray results (see Fig. 5b and c).

**Fig. 8:**
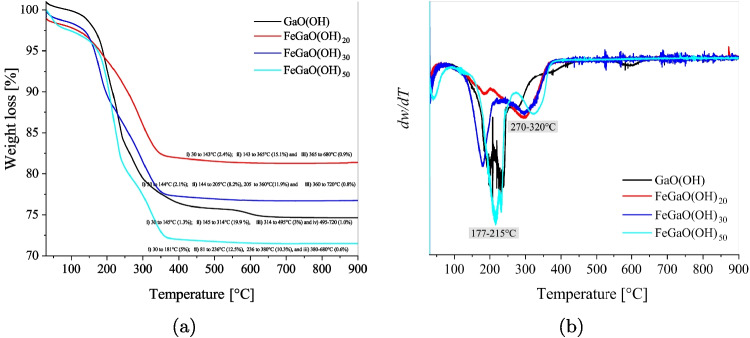
**a** Weight loss and **b** weight derivative as a function of the sample temperature

TGA analysis of the gallium-iron-based materials was carried out under a nitrogen atmosphere, from ambient temperature to 950 $^{\circ }\text {C}$. Figure 8 shows the weight percentage loss and weight derivative as a function of the sample temperature for GaO(OH), FeGaO(OH)_20_, FeGaO(OH)_30_, and FeGaO(OH)_50_. The thermal analysis of Fe-Ga oxyhydroxide materials (see Fig. 8) showed a weight loss in the TGA curve and peaks in weight derivative curves varying in the range from 150 to 400 $^{\circ }\text {C}$ (depending on the relation Ga:Fe). The thermal dehydroxylation of gallium-iron-based materials leads to this behavior, while the iron content shifts the curves.

**Table 5:** Deconvolution O1*s* and Ga3*d* for GO photocatalyst

		GO		
Orbital	Functional	Position	FWHM	Relative
	group	eV	eV	content (%)
Ga3*d*	Elemental Ga	18.26	2.43	30.5
	Ga$^{+3}$	19.75	1.70	40.5
	Ga$_x$O$_y$	20.63	3.09	29.0
O1*s*	Oxides	529.31	2.88	29.1
	O$_{(1)}$	530.41	1.20	41.8
	O$_{(2)}$	532.00	1.21	17.4
	O$_{(3)}$	532.99	3.12	11.8

**Table 6:** Deconvolution for the different Fe-doped $\beta$-Ga_2_O_3_ photocatalysts

Orbital	Functional group	Position (eV)	FWHM (eV)	Relative content (%)
		**FeGO**_**20**_		
Ga3*d*	Elemental Ga	18.26	1.41	11.9
	Ga$^{+3}$	19.53	1.90	67.3
	Ga$_x$O$_y$	21.42	4.14	20.8
O1*s*	Oxides	529.47	1.79	23.4
	O$_{(1)}$	530.13	1.58	44.1
	O$_{(2)}$	531.06	1.34	19.8
	O$_{(3)}$	532.06	1.91	12.7
Fe2$p_{3/2}$	Fe	710.67	3.98	74.1
	Fe$^{+2}$	713.82	2.14	14.8
	Fe$^{+3}$	715.78	2.32	11.1
		**FeGO**_**30**_		
Ga3*d*	Elemental Ga	19.09	1.42	16.2
	Ga$^{+3}$	19.91	1.59	52.3
	Ga$_x$O$_y$	20.93	4.34	31.5
O1*s*	Oxides	528.64	2.42	4.4
	O$_{(1)}$	530.21	1.56	48.7
	O$_{(2)}$	531.10	2.08	41.8
	O$_{(3)}$	532.85	2.27	5.0
Fe2$p_{3/2}$	Fe	709.36	4.14	27.0
	Fe$^{+2}$	711.30	3.59	48.9
	Fe$^{+3}$	714.52	4.03	24.1
		**FeGO**_**50**_		
Ga3*d*	Elemental Ga	19.57	1.70	33.2
	Ga$^{+3}$	20.61	1.55	41.4
	Ga$_x$O$_y$	21.91	2.29	25.3
O1*s*	Oxides	530.19	1.66	29.7
	O$_{(1)}$	531.11	1.46	31.5
	O$_{(2)}$	532.16	1.60	25.1
	O$_{(3)}$	533.43	2.13	13.7
Fe2$p_{3/2}$	Fe	709.77	2.31	24.6
	Fe$^{+2}$	711.62	2.13	34.6
	Fe$^{+3}$	713.91	3.46	40.8

From the weight loss plot (see Fig. 8a), it is possible to distinguish four regions whose limits are: (i) $145 - 150^{\circ }$C, (ii) $210-250^{\circ }$C, and (iii) $340-350^{\circ }$C, depending on the material. These thresholds set a behavior change. In Zone A (from ambient temperature to $145 - 150^{\circ }$C), the weight loss ($\approx 1.3-2.4\%$) is associated with the superficial water. In Zone B (from $145 - 150$ to $210-250^{\circ }$C), the weight loss ($\approx 7.5-13.5\%$) is related to the loss in the adsorbed water in the structure. The detachment of hydroxyl groups, indicating the elimination of the adsorbed moisture while the material is exposed to a nitrogen atmosphere, happens in Zone C (from $210-250$ to $340 - 350^{\circ }$C) and represents a loss of about $9-10\%$. Finally, the crystallization process occurs in Zone D (from $340 - 350$ to $900^{\circ }$C), with a weight loss below $1\%$. In this zone, a transition from the $\alpha$ to the $\beta$ phase of Ga_2_O_3_ can be observed around $620^{\circ }$C.

Figure 8b shows the weight derivative plots. The thermal behavior is consistent with reported gallium-based materials (Orozco et al. 2023). In this case, it is possible to distinguish two endothermic peaks, which are attributed to the hydroxyl groups elimination processes. Furthermore, it can be observed that the endothermic peak located around 271 $^{\circ }\text {C}$ for GaO(OH) shifts towards a higher temperature as Fe content increases, reaching 329 $^{\circ }\text {C}$ for FeGaO(OH)_50_.

**Fig. 9:**
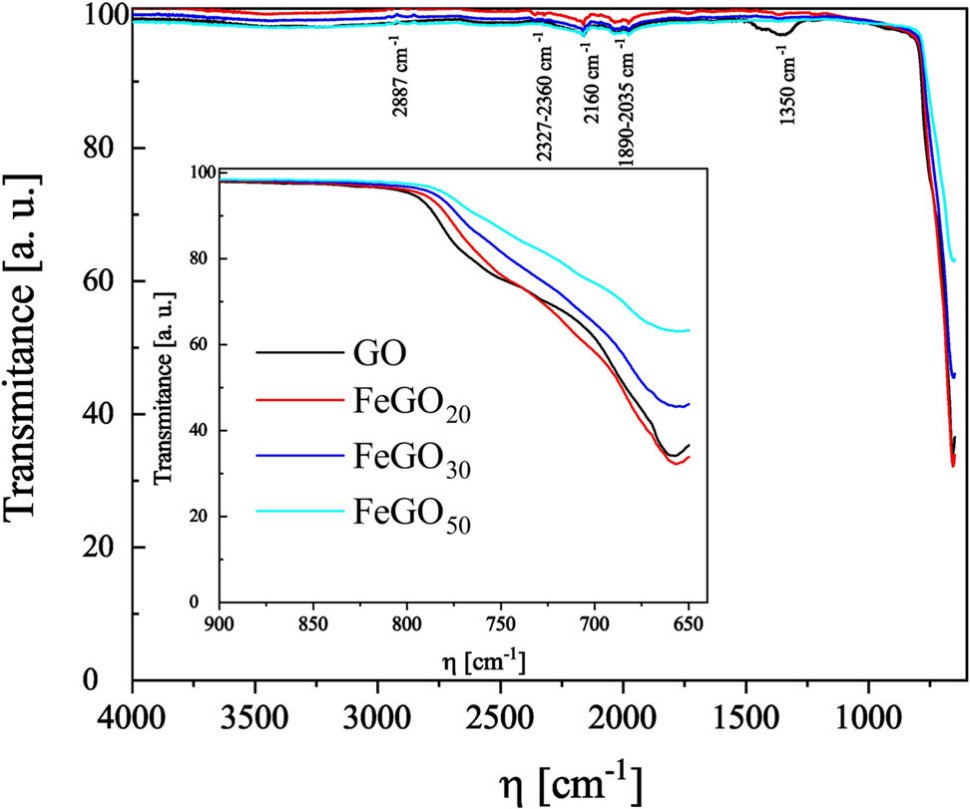
IR Spectra of gallium oxide doped with Fe (FeGO_20_, FeGO_30_, and FeGO_50_) treated at ${950\,\mathrm{{ ^{\circ }\text {C}}}}$

The FT-IR spectra for the gallium-based photocatalysts GO, FeGO_20_, FeGO_30_, and FeGO_50_ (synthesized at pH 10 and heat treatment at ${950\,\mathrm{{ ^{\circ }\text {C}}}}$) are presented in Fig. 9. The FT-IR spectra of all materials reveal an OH stretching band at around 3395 cm$^{-1}$, corresponding to adsorbed water molecules. The band at 1890 cm$^{-1}$ can be assigned to constitutional Ga-OH bending bands and their overtones. The stretching vibration bond of the O-H group is also observed around 1350 cm$^{-1}$ owing to the absorption of water molecules (Quan et al. 2010). In addition, the four photocatalysts show a band at 650 to 770 cm$^{-1}$, which is assigned to the valence vibrations of Ga-O in the lattice formed by GaO_4_ tetrahedral, corresponding to monoclinic structures of $\beta$ crystalline phases, respectively. These results are consistent with those reported by Quan et al. (2010); Yang et al. (2009); Girija et al. (2013); Varella Rodrigues and Sabino (2019). In FT-IR spectra for FeGO_20_, FeGO_30_, and FeGO_50_, species associated with Fe ions are not identified. Moreover, the FT-IR spectrum for Fe doped photocatalysts exhibits wider bands caused by a large number of IR-active modes due to a lower symmetry of FeGO$_{x}$ and Fe_2_O_3_ identified in XRD and XPS (Figs. 5 and 7) (Neumann et al. 2011). The increase of Fe in the gallium-based photocatalysts induces a shift in the IR bands position to higher wave numbers, with respect to GO (samples having the monoclinic crystal structure).

**Fig. 10:**
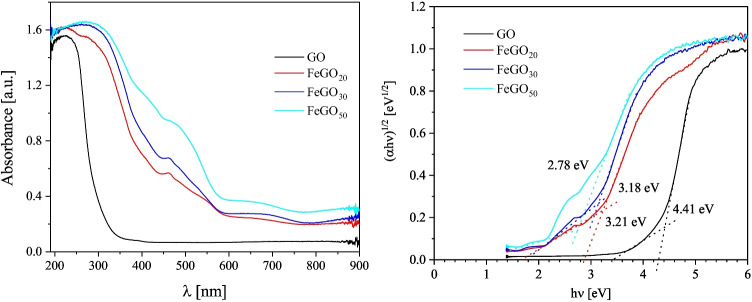
**a** UV–Vis diffuse absorbance spectra and **b** $(\alpha h \nu )^{1/2}$ versus $h \nu$ plots for FeGO_20_, FeGO_30_, and FeGO_50_ synthesized by alkaline precipitation assisted with ultrasonic transducer and treated at 950 $^{\circ }\text {C}$

The optical properties of gallium oxides and Fe-doped gallium oxides were determined by UV–Vis spectroscopy in diffuse absorbance mode. The diffuse absorbance spectra for GO, FeGO_20_, FeGO_30_, and FeGO_50_ are shown in Fig. 10. For GO, an absorption band centered at 250 nm in the spectrum of $\beta$-Ga_2_O_3_ corresponds to the O-Ga charge transfer transition (Nikolaev et al. 2019). FeGO_20_, FeGO_30_, and FeGO_50_ photocatalysts show new absorption bands in the UV–visible region at 294, 460, and 680 nm. The spectra of iron(III) oxides in the near-infrared, visible, and ultraviolet regions are due to transitions between the energy states of electrons located in outer atomic orbitals, which are involved in the formation of chemical bonds in these compounds (3*d* electrons of Fe and 2*p* electrons of O) (see Fig. 7b, c, and d). For FeGO_30_ and FeGO_50_, the absorption band at 294 nm corresponds to iron oxide, also identified in XRD spectra (see Fig. 5b and c) (Gurgul et al. 2013). A steep increase in absorption (absorption edge) takes place between about 400 and 500 nm, and the inflection point of this curve (at $\approx 480$ nm) corresponds to the Fe_2_O_3_ band gap energy ( $\approx 2$ eV) (Al-Kuhaili et al. 2012; Gurgul et al. 2013; Tahir et al. 2021). In the visible region of the spectrum, an absorption band in the 600–750 nm range is due to impurities for all Fe-doped systems. This absorption band occurs by the incorporation of Fe ions, which provide $3\textit{d}$ molecular orbitals, favoring intraband electronic transitions between the electronic states of O$2\textit{p}$ and the $3\textit{d}$ metal ions states. These can be inferred from the density of states and the extinction coefficient described in the computational section.

On the other hand, the fundamental absorption can be observed, which shifts towards longer wavelengths as the Fe concentration increases, accentuating the importance of adjusting the bandgap with metal ions. These results are consistent with those observed in the XRD patterns and XPS spectra, Figs. 5 and 6, respectively.

**Fig. 11:**
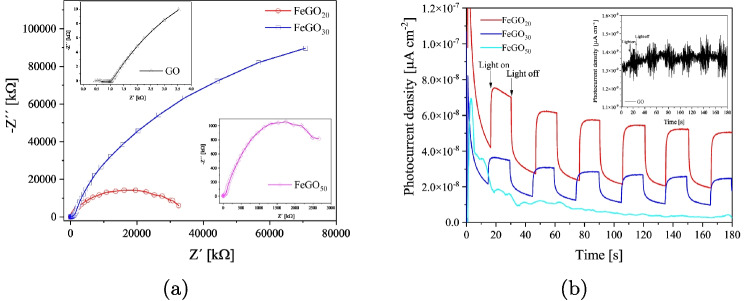
**a** EIS spectra and **b** transient photocurrent responses of GO, FeGO_20_, FeGO_30_, and FeGO_50_ photocatalyst

The UV–Vis spectra of GO, FeGO_20_, FeGO_30_, and FeGO_50_ photocatalyst were used to calculate their band gap by the Tauc plot method, Eq. 15:

15 $$\begin{aligned} \left( \alpha h \nu \right) ^{1/2} = k \left( h \nu -E_{g} \right) \, , \end{aligned}$$

where $\alpha$ is the absorption coefficient, *k* is the parameter related to the effective masses associated with the valence and conduction bands, $h \nu$ is the absorption energy, and $E_{g}$ is the band gap energy.

According to the Tauc plot method, the extrapolated intercept of the $\left( \alpha h \nu \right) ^{1/2}$ variation with $\textrm{h} \nu$ gives the corresponding $E_{g}$ value. The band gap of GO is 4.41 eV, corresponding to UV light absorption (281 nm). This value agrees with values reported in literature by Liu et al. (2018); Michel and Martínez-Preciado (2022a). After Fe doping, the band gap of FeGO_20_, FeGO_30_, and FeGO_50_ decreases to 3.21, 3.18, and 2.78 eV, respectively. Proving that Fe doping promotes an absorption band FeGO$_{x}$ shift via the formation of Fe_2_O_3_ in FeGO_20_, FeGO_30_, and FeGO_50_ (Zeng et al. 2023; Hany et al. 2019).

It is worth mentioning that both experimental and theoretical $E_g$ values exhibit the same tendency: the higher the Fe content, the lower the band gap energy. For the GO and 50%Fe:$\beta$-Ga_2_O_3_ photocatalysts (see Table 1), both results are in good agreement. Moreover, the $E_g$ value for GO is consistent with reported values (Rappe et al. 1990). In turn, the $E_g$ values for FeGO_20_ and FeGO_30_ are lower than the theoretical values determined for 12.5%Fe:$\beta -$Ga_2_O_3_ and 25%Fe:$\beta -$Ga_2_O_3_. This can be attributed to the fact that the theoretical values were assessed using parameters for materials with lower theoretical iron content, in contrast to the theoretical content considered in this work and the real content incorporated in the gallium oxide matrix.

The EIS spectra shown in Fig. 11 have a semi-arch shape for FeGO_20_, FeGO_30_, and FeGO_50_, which represents the rate of electron transmission. The smaller the Nyquist arc radius, the greater the electron transfer and vice versa (Parida et al. 2023). From Fig. 11a, it can be observed that the semicircle shape becomes more marked (smaller Nyquist arc radius) with an increase in Fe ion incorporation, indicating faster charge transfer processes for FeGO_20_. Conversely, the more heterogeneous behavior for FeGO_30_ and FeGO_50_ corresponds to slower charge transfer processes (lower impedances). The charge transfer efficiency was further evaluated using transient photocurrent responses, as shown in Fig. 11b. FeGO_20_ photocatalysts recorded the highest photocurrent density ($7.5\times 10^{-8} \mu$A cm$^{-2}$), about 5 times higher than that of the undoped $\beta -$Ga_2_O_3_ semiconductor. Thus, adding Fe ion in Ga_2_O_3_ introduces energetic levels that favor the presence of charge carriers for the case of FeGO_20_ and FeGO_30_. While for a 50% Fe content, the high heterogeneity reduces the efficiency of the photo-response. Therefore, FeGO_20_ has higher light absorption capacity, reduced recombination of photoinduced charge carriers, and enhanced electron transfer.

**Fig. 12:**
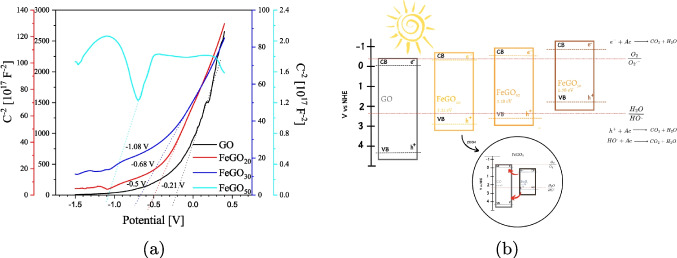
**a** Mott-Schottky plots of GO, FeGO_20_, FeGO_30_, and FeGO_50_ photocatalyst at 1 kHz, and **b** proposed band diagram

In addition, Mott-Schottky (M-S) measurements were carried out and shown in Fig. 12a, it can be observed that the capacitance decreases as the value of 1/C$^2$ increases. This indicates that the FeGO_20_ solution presents a higher polarizability of the medium, which is consistent with the theoretical results (see “Numerical results” section), where the dielectric function plot (see Fig. 4) also shows that the best polarizability corresponds to the lowest doping concentration. On the other hand, the positive slope observed for GO, FeGO_20_, and FeGO_30_ demonstrates their semiconductor type-*n* character. In Fig. 12a, an irregular slope is evident for FeGO_50_ photocatalyst. This behavior is associated with the heterogeneity of the system, so it is possible to appreciate the regions of accumulation ($-1.5$ to $-1.1$ V), depletion ($-1.1$ to $-0.7$ V), and inversion (above $-0.7$ V). In addition, Fig. 12 shows the existence of several potential regions characterizing the semiconducting behavior of the corresponding system. In the potential region between $-$1.5 V and $-$0.7 V, all doped systems exhibit a *p*-type behavior, while for the region between $-$0.7 and 0.2 V, the behavior is *n*-type. The progressive shift towards negative values of V$_{fb}$ suggests that doping with Fe modifies the alignment of bands, bringing the Fermi level closer to the conduction band. This behavior is more evident for FeGO_30_ and FeGO_50_, possibly due to phase segregation, as suggested by the theoretical modeling (see “Density of states” section).

From the M-S plots, it is possible to determine the V$_{fb}$ of the materials from the intersection obtained by extrapolating the linear-region line and the *x*-axis (potential) (Parida et al. 2023). V$_{fb}$ for GO, FeGO_20_, FeGO_30_, and FeGO_50_ photocatalyst were $-0.21$, $-0.5$, $-0.68$, and $-1.08$ V, respectively. Then, the VB potential ($V_{VB}$) can be calculated from Eq. 2. The band configuration of GO, FeGO_20_, FeGO_30_, and FeGO_50_ is depicted in Fig. 12b. It can be inferred that incorporating Fe ions shifts the $V_{VB}$ potentials, enabling visible light harvesting, altering the degradation mechanism via radicals, and influencing the photocatalytic activity (Lee et al. 2024). For FeGO_30_, $E_{CB}$ and $E_{VB}$ were above the O$_2$/O_2_^–•^ standard potential and below that of H_2_O/HO^•^ (see Fig. 12b), respectively. This allows photocatalytic activity to occur in both the conduction and valence bands. Nevertheless, for GO and FeGO_x_, the photocatalytic activity could happen at the valence band (H_2_O/HO^•^), while for FeGO_50_ at the conduction band (O$_2$/O_2_^–•^). Fe$^{2+}$ and Fe$^{3+}$ species play a crucial role in facilitating charge transfer processes, acting as an internal redox pair that can promote electron–hole separation and reduce recombination rates.

The morphology and microstructure of FeGO$_{x}$ photocatalysts were studied by SEM, as shown in Fig. S6 in the SM. The GO photocatalyst shows nanorod shapes with characteristic lengths and widths of $500-700$ nm and $160-220$ nm, respectively (see Fig. S6(a) in SM). This morphology is consistent with observations by Orozco et al. (2023), in which gallium oxides, based on gallium liquid metal, were doped with Cu and synthesized by the ultrasonic transducer method. Slightly smaller dimensions are observed for FeGO_20_, except that this photocatalyst (and all remaining Fe-doped materials) exhibit voids whose dimensions are $\sim$ 20 nm (see Fig. S6(b) in SM). This indicates that incorporating Fe in the gallium oxide matrix leads to the growth of cavities towards the core of the GO material. Similar results were reported by Kang et al. 2022. When the Fe content increases (for FeGO_30_ and FeGO_50_ photocatalyst), the morphology collapses, forming agglomerates of more irregular nanospindles structures with smaller dimensions and voids content (see Fig. S6(c) and (d) in SM). N$_{2}$ adsorption/desorption study of the GO and FeGO$_{x}$ photocatalysts showed that the photocatalysts are nonporous with BET surface area of 8.52 to 7.29 m^2^g^–1^, where the increase of Fe content in Ga_2_O_3_ slightly decreased the surface area.

**Fig. 13:**
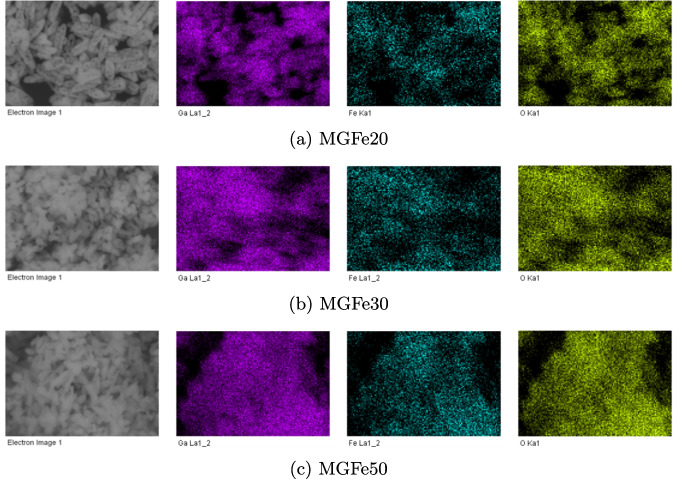
SEM for **a** $20\%$, **b** $30\%$, and **c** $50\%$ Fe doping

The elemental mapping of FeGO_20_, FeGO_30_, and FeGO_50_ photocatalysts is shown in Fig. 13 and is resumed in Table 4. As observed, the distribution of Fe, Ga, and O is similar for all Fe-doped gallium oxides (see Fig. 13). SEM-EDS spectra for FeGO_x_ materials are shown in Fig. S7 in the SM. The atomic ratio of Ga:Fe is 86.74:13.26, 76.51:23.79, and 80.88:19.12 for FeGO_20_, FeGO_30_, and FeGO_50_, respectively (see Table S2 in SM).

TEM images of the synthesized materials are shown in Fig. S8 in the SM. It is evident that the pristine system and the doped one, with a concentration of 20% iron, present nanorod-type shapes. The average length of the nanorods (see Fig. S8(a) in the SM) is 811 nm, with a diameter of 275 nm, while the average length of the nanorods for the phase with a concentration of 20% Fe is 460 nm and a diameter of 166 nm. Notably, with increasing Fe concentration, the size of the nanorods is reduced, obtaining agglomerates of irregular particles with an average radius of 126 nm and 77 nm for concentrations of 30% and 50% Fe, respectively. The reduction in nanorod size can be caused by the increase in iron content, whose *d* orbitals increase their interaction with oxygen orbitals, which is reflected in the bond length between Fe-O and Ga-O (see DFT analysis in “Numerical results” section).

**Fig. 14:**
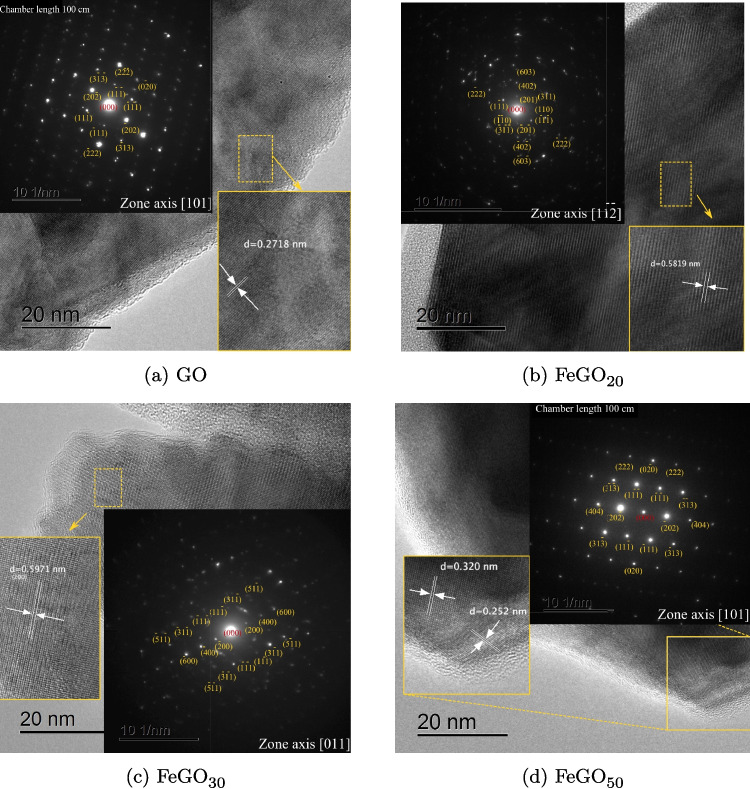
The high-resolution transmission electron microscope (HRTEM), SAED pattern, and interplanar spacing of **a** GO, **b** FeGO_20_, **c** FeGO_30_, and **d** FeGO_50_. The colored box was used to obtain the fast Fourier transform and determine the interplanar space

High-resolution TEM (HRTEM) allows direct imaging of the atomic layers of these nanoparticles and the subsequent identification of the crystal lattice structure through fast Fourier transform (FFT) analysis. The 20 nm scale TEM images are shown in Fig. 14. From these images, the interplanar separations were measured, being consistent with the corresponding XRD observed crystalline planes. Based on the SAED standard of $\beta$-Ga_2_O_3_, it is confirmed that both nanorods (Fig. 14a and b) and agglomerated particles (Fig. 14c and d) present the desired phase with multiple reflection peaks, demonstrating the polycrystalline nature of the synthesized samples and preserving the characteristic planes of the $\beta$ phase, even with the incorporation of Fe. Zone axis and corresponding indexing have been added to SAED images.

### Photocatalytic test

This section aims to evaluate the impact of employing GO, FeGO_20_, FeGO_30_, and FeGO_50_ photocatalysts in the photocatalytic degradation of the emerging model pollutant acetaminophen.

In the photodegradation analysis of any pollutant, photolysis is essential because it establishes to which extent light-induced reactions, independent from other oxidative or catalytic processes, contribute to its degradation. Namely, it is important to quantify photolysis to prevent it from hindering photocatalysis. The results of the Ac photolysis show that Ac photodegradation is minimal under UVA ($\approx 8$%) and visible ($\approx 1$%) illumination conditions, confirming that the degradation process occurs via the photocatalytic process.

The adsorption of Ac on the Ga_2_O_3_ particles surface is a very important step in photocatalytic degradation. Adsorption time for Ac was investigated with Ga_2_O_3_ for pH 5, 0.5 g L^–1^ of catalyst, and 12 mg L^–1^ of Ac under dark condition. The experimental results showed that full adsorption was achieved within 30 min, reaching 18% of molecules of Ac adsorbed on the surface of Ga_2_O_3_ (see Fig. S9 in SM). After this time, the desorption process begins, as reported by Lee et al. (2024). Therefore, in all subsequent experiments, a period of 30 min of adsorption in the dark was allowed before starting irradiation. This time was set as $t =0$ min.

The experimental conditions were determined by a three-way factorial design $\left( 3\times 3 \right)$ considering GO as the catalyst. The dependent variable corresponds to acetaminophen (Ac) degradation, while the independent variables and corresponding levels are pH (3, 5, 7, and 9), acetaminophen concentration (6, 12, and 24 mg L$^{-1}$), and GO concentration (0.5, 0.75, and 1 g L^–1^). We use the optimum oxidizing agent concentration of $1.2\times 10^{-5}$ M (Gholami et al. 2021; Orozco et al. 2023) to investigate the photocatalytic Ac degradation. To study the effect of pH, a GO concentration of 0.5 g L^–1^ and 12 mg L^–1^ of Ac were used; for the Ac concentration we used a GO concentration of 0.5 g L^–1^ and pH of 5; and for the catalyst concentration, a pH 5 and 12 mg L^–1^ of Ac were employed.

**Table 7:** Comparison of the pseudo-first-order kinetic constants, $k_{1}$ in photodegrading acetaminophen using gallium-based photocatalysts and other materials

Photocatalyst	Conditions	$k_{1}$ [min$^{-1}$]	Comments	Ref
GO	pH $3-9$, $C_{Cat}=0.5-{1.0}\,\text {g}\,\text {L}^{-1}$, $C_{Ac}=6-{24}\,\text {mg}\,\text {L}{-1}$, **UVA Lamp (320–400 nm, 2.87 W)**	0.000153–0.004867	Optimal parameters: pH 5, $C_{Ac}=$ 12 mg L^–1^ and $C_{Cat}=$ 0.5 g L^–1^	This work
FeGO_x_	(x$=20$, 30 and 50% wt) $C_{Cat}= {0.5}\,\text {g}\,\text {L}^{-1}$, $C_{Ac}={12}\,\text {mg}\,\text {L}{-1}$, **UVA Lamp (320–400 nm 2.87 W)**	0.00653–0.00763	5-cycle stability study, $k_1$ varies $\sim 20\%$ through cycling, FeGO_20_ ($k_1=$0.00653–0.01008 min$^{-1}$), FeGO_30_ ($k_1=$0.00536–0.00933 min$^{-1}$), FeGO_50_ ($k_1=$0.00711–0.00828 min$^{-1}$)	This work
FeGO_x_	(x$=20$, 30 and 50% wt) $C_{cat}= {0.5}\,\text {g}\,\text {L}^{-1}$, $C_{Ac}= {12}\,\text {mg}\,\text {L}^{-1}$, **Visible Lamp (400–700 nm, 2.25 W.)**	0.00324–0.00562	5-cycle stability study, $k_1$ varies $\sim 20\%$ through cycling, FeGO_20_ ($k_1=$0.00189–0.00324 min$^{-1}$), FeGO_30_ ($k_1=$0.00545–0.00678 min$^{-1}$), FeGO_50_ ($k_1=$0.00333–0.00513 min$^{-1}$)	This work
Cu_x_GO	(x=5, 7 and 10% wt) $C_{cat}={0.5}\,\text {g}\,\text {L}^{-1}$, $C_{Ac}=$12 mg L^–1^, **Visible Lamp (400–700 nm)**	$k_1=$0.00645, 0.00486 and 0.00153 min$^{-1}$ for 5, 7 and 10% wt, respectively	Stable after five cycles	(Orozco et al. 2024, 2023)
Cu_x_/Ga_2_O_3_/In_2_O_3_	(x=5, 7 and 10% wt) $C_{cat}= {0.5}\,\text {g}\,\text {L}^{-1}$, $C_{Ac}=$ 12 mg L^–1^, **Visible Lamp (400–700 nm)**	$k_1=$0.006551±0.000241, 0.005832±0.000069 and 0.003980±0.000006 min$^{-1}$ for 5, 7 and 10% wt, respectively	5-cycle stability study, significant reduction in photocatalytic activity after the first cycle	(Murguía et al. 2025)
TiO_2_ DP25	$C_{cat}= 0.06-{0.4}\,\text {g}\,\text {L}^{-1}$, $C_{Ac}= 10- {100}\,\text {mg}\,\text {L}^{-1}$, **UVC Lamp (254 nm)**	$k_{Ap}=$0.1125 min$^{-1}$ (Absorbed irradiation =23,523 W m$^{-2}$)	Evaluation of the local volumetric rate of energy absorption for different experimental conditions	(Alvarado-Rolon et al. 2021)
Ag/TiO_2_	Ag_x_/TiO_2_ ($x=2-8$% wt), $C_{cat}= {0.25}\,\text {g}\,\text {L}^{-1}$, $C_{Ac}= {17}\,\text {mg}\,\text {L}^{-1}$, **UV Lamp** ($\lambda$ **emission not available**)	0.018–0.022 (Ag/TiO_2_) and 0.025 (TiO_2_ P25)	Photocatalytic activity decreases after the first cycle, reaching 63% removal in the fourth cycle	(Lee et al. 2024)
FTiO_2_-350, FTiO_2_-500	FTiO_2_-350, FTiO_2_-500 $C_{cat}=$ 0.25 g L^–1^, $C_{Ac}=$ 5 mg L^–1^, Xe lamp (**320 nm**, **600 W m**$^{-2}$)	0.020–0.048	After five cycles, no decrease in catalytic activity was observed for FTiO_2_-350, while FTiO_2_-350 decreased 20%	(García-Rollán et al. 2025)
Zn-Ag co-doped barium hexaferrite-CNTs Composite	$C_{cat}=$ 0.42 g L^–1^, $C_{Ac}=$ 10 mg L^–1^, **Xenon lamp (350 a 1100 nm)**	0.005–0.016	The photocatalytic activity remains active for 6 cycles with $\approx 2\%$ loss in efficiency	(Irshad et al. 2024)
Ag/ZnO	$C_{cat}=0.05- {0.25}\,\text {g}\,\text {L}^{-1}$, $C_{Ac}= 1-{9}\,\text {mg}\,\text {L}^{-1}$, pH=1-9, **UV Lamp (8 W)**	0.06026	Optimal parameter: pH$=$11, $C_{cat}=$0.15 g and $C_{Ac}=$5 g L$^{-1}$. Ac degradation efficiency decreased 6% after 5 cycles	(Mohsentabar et al. 2023)
MOFs UiO-66-NH$_2$ UiO-66-NH-C$_6$ UiO-66-NH-C$_5$	$C_{cat}= {0.25}\,\text {g}\,\text {L}^{-1}$, $C_{Ac}= {5}\,\text {mg}\,\text {L}^{-1}$, pH=4, **Xe Lamp** (**over 320 nm, 600 W m**$^{-2}$)	0.013–0.090	The photocatalyst shows $\approx 10\%$ loss in the activity after 5 cycles	(Gómez-Avilés et al. 2023)
K$_3$[Fe(CN)$_6$]/TiO$_2$, TiO_2_-P25	$C_{cat}= {1}\,\text {g}\,\text {L}^{-1}$, $C_{Ac} = {15}\,\text {mg}\,\text {L}^{-1}$, **Visible Lamp (LED 450–550 nm)**	0.00749 (K$_3$[Fe(CN)$_6$]/TiO$_2$), 0.00162 (TiO_2_-P25)	Study of optimal parameters: pH, $C_{Ac,0}$, $C_{cat}$, temperature, and number of lamps	(Gotostos et al. 2014)
Immobilized TiO_2_ P25	$C_{cat}= {0.28} \text {g}$, $C_{Ac}= 5-{65}\,\text {mg}\,\text {L}^{-1}$, **Solar simulator** (**280–400 nm, 450 W m**$^{-2}$)	0.028–0.0028	Optimal conditions:$C_{Ac,0}$ and $C_{cat}$. 6-cycle stability study, the activity photocatalytic remains through all cycles	(Yahiaoui et al. 2024)
TiO_2_ nanotubes	$C_{cat}= {0.4}\,\text {g}\,\text {L}^{-1}$, $C_{Ac}= {10}\,\text {mg}\,\text {L}^{-1}$, **UVC Lamp (254 nm)**	0.021–0.03	Identification of intermediates during photocatalytic decomposition	(Lopez Zavala and Delgado Juárez 2024)
TiO_2_	$C_{cat}={2}\,\text {g}\,\text {L}^{-1}$, $C_{Ac}=80-{160}\,\text {mg}\,\text {L}^{-1}$, **4 UV lamps (365 nm)**	$k_1=$ 0.001735 and $k_2=$0.004037	Identification of intermediates during photocatalytic decomposition	(Aguilar et al. 2022)
Cu_2_O/WO_3_/TiO_2_	$C_{cat}= {0.25}\,\text {g}\,\text {L}^{-1}$, $C_{Ac}= {10}\,\text {mg}\,\text {L}^{-1}$, **Xenon lamp (350–1100 nm)**	0.0442	Optimal conditions: $C_{Ac,0}$ and $C_{cat}$. 5-cycle stability study, the activity photocatalytic remains in all cycles	(Chau et al. 2022)
Fe-g-C_3_N_4_	$C_{cat}= 0-{0.3}\,\text {g}\,\text {L}^{-1}$, $C_{Ac}= {50}\,\text {mg}\,\text {L}^{-1}$, **Dark conditions**	–	4-cycle stability study: the photocatalytic activity remains active after each cycle with approximately 5% loss in efficiency	(Mu et al. 2025)
TiO_2_/NiO$_x$ nanofibers	x=1, 3 and 5% wt., $C_{cat}=$ 0.5 g L^–1^, pH$=7$, $C_{Ac}= {10}\,\text {mg}\,\text {L}^{-1}$, **Halogen Visible Lamp** (**400 W, 318 mW cm**$^{-2}$)	0.0032–0.0133	5-cycle stability study: the photocatalytic activity exhibited a progressive decrease	(Abid et al. 2025)
Mg$_{0.4}$Zn$_{0.6}$FeO_3_, Mg$_{0.4}$Zn$_{0.6}$- FeO_3_/CNTs	$C_{Cat}=$ 0.42 g L^–1^, $C_{Ac}=$ 25 mg L^–1^, **Tunsten Visible Lamp (200 W)**	0.00878–0.01796	4-cycle stability study: the photocatalytic activity remains active after each cycle with approximately 5% loss in efficiency	(Moin et al. 2025)
PbBiO_2_Br, Pt/PbBiO_2_Br/MnO_2_-(Co_3_O_4_)	$C_{Cat}=$ 1 g L^–1^, $C_{Ac}=$ 15 mg L^–1^, **Xenon Lamp (300 nm, 300 W)**	0.0015–0.00858	The photocatalyst remains active after three cycles	(Dong et al. 2025)

The Ac photocatalytic degradation profiles with GO under UVA illumination and different experimental conditions are shown in Fig. S10 (see SM). The optimal Ac degradation (78%) is achieved at pH 5, which can be explained by the electrostatic interaction between the catalyst surface (GO isoelectric point of 4.0 Orozco et al. 2023) and the ionization of the Ac molecule. As for Ga_2_O_3_ concentration, the higher degradation ($78\%$) is obtained with the lower catalyst concentration (0.5 g L^–1^) due to the optical path shortening within the reaction space with increasing catalyst concentration. Hence, fewer photons are available to activate the catalyst, resulting in fewer reactions (Orozco et al. 2022b). In turn, the higher the Ac concentration, the longer the reaction time required to degrade the higher number of pollutant molecules, although no significant difference was observed for Ac concentrations of 6 and 12 mg L^–1^. Figure S10 (see SM) also shows the experimental results fitted to the pseudo-zero and pseudo-first-order kinetic models for the explored conditions. The corresponding pseudo kinetic constants, $k_{0}$ and $k_{1}$, are presented in Table S3 in the SM. The coefficients of determination close to unity indicate a good fit. No significant differences are observed in the models, indicating that the Ac degradation rate does not influence the Ac concentration under the explored conditions. It is important to mention that $k_1$ are in the same order of magnitude as those values reported for FeCo-MOF (0.0082 min$^{-1}$) (Pattappan et al. 2023), Zn-Ag co-doped barium hexaferrite-CNTs composite (0.005 min$^{-1}$) (Irshad et al. 2024), K_3_[Fe(CN)_6_]/TiO_2_ (0.00749 min$^{-1}$), TiO_2_/NiO_x_ nanofibers (0.0032$-$0.0133 min$^{-1}$) (Abid et al. 2025), Mg$_{0.4}$Zn$_{0.6}$FeO_3_- Mg$_{0.4}$Zn$_{0.6}$FeO_3_/CNTs (0.00878$-$0.01796 min$^{-1}$) (Moin et al. 2025), PbBiO_2_Br - Pt/Pb-BiO_2_Br/MnO_2_-(Co_3_O_4_) (0.0015$-$0.00858 min$^{-1}$) (Dong et al. 2025). These values were obtained under similar conditions or even with higher-power light sources than those used in the present work. In Table 7, different photocatalysts are compared with those reported in this work. Other authors reported $k_1$ values an order of magnitude higher ($0.020-0.079$ min$^{-1}$) corresponding to TiO_2_-based photocatalysts (Chau et al. 2022; Dudziak et al. 2023; Lopez Zavala and Delgado Juárez 2024). It is worth noting two aspects for some of these TiO_2_-based photocatalysts: (i) lamps with higher energy and power were used (UVB-UVC, $\approx 200-300$ W) compared to the low-power (UVA, $\approx 10$ W) used in this work, and (ii) smaller volumes were considered. Therefore, higher degradation rates are expected under these conditions. GO did not show photocatalytic activity under visible illumination and the explored conditions.

**Fig. 15:**
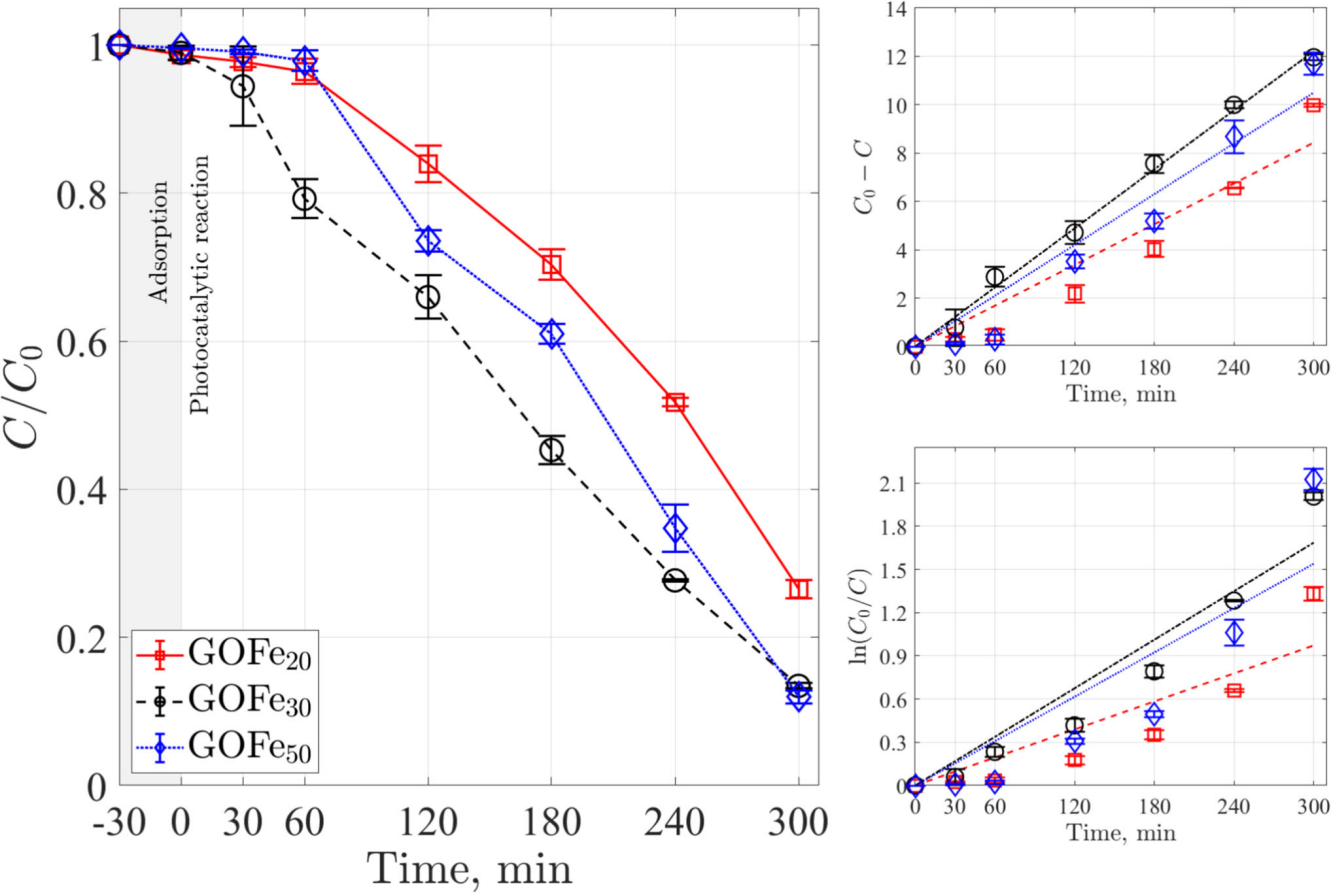
*Left*) Photocatalytic degradation of acetaminophen with FeGO_x_ at pH 5, 0.5 g L^–1^ of catalyst and 12 mg L^–1^ of Ac under visible illumination. (*Top-right*) Pseudo-zero-order fitting. (*Bottom-right*) Pseudo-first-order fitting

According to these results, the conditions to study the photocatalytic activity of the FeGO_20_, FeGO_30_, and FeGO_50_ are as follows: pH 5, and a photocatalyst (FeGO_x_) and acetaminophen concentrations of 0.5 g L^–1^ and 12 mg L^–1^,

respectively. The degradation profiles of acetaminophen under visible illumination are shown in Fig. 15, evidencing their photocatalytic activity under visible illumination by the decrease in the energy band when doping Fe ions. These experimental results were fitted to pseudo-zero-order and pseudo-first-order models, Eqs. 6 and 7, and the corresponding plots are presented in Fig. 15. The degradation efficiency of FeGO_x_ under visible light is comparable to or even higher than that of GO under UVA light (see Fig. S10 in SM). This aligns with the results from EIS and DFT, which indicated that incorporating iron ions into the gallium oxide matrix promotes an absorption band shift of the catalyst towards lower values, thereby enhancing the material’s degradation performance (Orozco et al. 2022a).

Several experiments of Ac degradation with FeGO_x_ under UVA light were carried out, and the results are shown in Fig. S11 in SM. Notably, high degradation percentages (up to $\sim 88\%$) were achieved at pH 5 under both visible and UVA light sources. Additionally, the Ac degradation profiles for the three photocatalysts exposed to UVA light exhibited similar trends, with some overlapping instances (see Fig. S11 in SM). It is important to note that when UVA light is used, an 80% degradation is obtained after 180 min. While under visible light, a degradation percentage between 80 and 88% is reached after 5 h.

**Table 8:** Pseudo-zero- and pseudo-first-order kinetic constants, $k_0$ and $k_1$, for FeGO_20_, FeGO_30_, and FeGO_50_ photocatalysts

Photocatalyst	$k_0$, L mg$^{-1}$ min$^{-1}$ (R$^{2}$)	
	UVA	Vis
FeGO_20_	$0.0409\pm 0.0008$ ($0.9701\pm 0.0015$)	$0.0281 \pm 0.0007$ ($0.9578\pm 0.0012$)
FeGO_30_	$0.0444\pm 0.0017$ ($0.9622\pm 0.0122$)	$0.0407\pm 0.0012$ ($0.9956\pm 0.0012$)
FeGO_50_	$0.0426\pm 0.0010$ ($0.9518\pm 0.0010$)	$0.0350\pm 0.0020$ ($0.9749\pm 0.0102$)
	$k_1$, min$^{-1}$ (R$^{2}$)	
FeGO_20_	$0.00653\pm 0.00083$ ($0.9249\pm 0.0008$)	$0.00324 \pm 0.00013$ ($0.8842\pm 0.0091$)
FeGO_30_	$0.00688\pm 0.00046$ ($0.9622\pm 0.0122$)	$0.00562\pm 0.00012$ ($0.9615\pm 0.0081$)
FeGO_50_	$0.00763\pm 0.00033$ ($0.9563\pm 0.0116$)	$0.00513\pm 0.00025$ ($0.8731\pm 0.0089$)

The experimental results for FeGO_20_, FeGO_30_, and FeGO_50_ under visible illumination show a better fit to a pseudo-zero-order model ($R{^2} \approx 1$), implying that the photocatalytic degradation only depends on the reaction time. The corresponding values of $k_0$ and $k_1$ for FeGO_20_, FeGO_30_, and FeGO_50_ under UVA and visible light are presented in Table 8. Nevertheless, the pseudo-first-order model results will be used to compare them with those reported in the literature. Note that the $k_1$ values do not change considerably under UVA light as the Fe content increases, exhibiting a monotonous increase. As for the pseudo-first-order kinetic constant under visible light, an increase is observed from FeGO_20_ to FeGO_30_, without significant variation for FeGO_50_. It is worth noting that our results with visible light ($k_1=$ 0.00324–0.00562 min$^{-1}$) are consistent with those reported for other materials such as K$_3$[Fe(CN)$_6$]/TiO$_2$ (0.00749 min$^{-1}$) (Gotostos et al. 2014), Zn-Ag co-doped barium hexaferrite-CNTs composite (0.005–0.016 min$^{-1}$) (Irshad et al. 2024), TiO_2_/NiOx (0.0032–0.0133 min$^{-1}$) (Abid et al. 2025), Mg$_0.4$Zn$_0.6$FeO_3_-Mg$_0.4$Zn$_0.6$FeO_3_/CNTs (0.00878–0.01796 min$^{-1}$) (Moin et al. 2025), Cu$_x$/Ga_2_O_3_/In_2_O_3_ ($0.003980-0.00655$ min$^{-1}$) (Murguía et al. 2025), which were obtained under similar experimental conditions (as can be observed in Table 7). As noted, when UVC light sources are used (implying high power and cost), $k_1$ increases considerably (by an order of magnitude) (Alvarado-Rolon et al. 2021; Chau et al. 2022; Mohsentabar et al. 2023; Lopez Zavala and Delgado Juárez 2024; Lee et al. 2024; Irshad et al. 2024), as could be expected.

The leached Fe from FeGO_20_, FeGO_30_, and FeGO_50_ photocatalysts after 300 min are $1.24\pm 0.02$, $1.07\pm 0.08$ and $1.15\pm 0.04$ mg L$^{-1}$, respectively. These Fe concentrations represent $\approx 0.5 - 1$%, depending on the FeGO_x_ photocatalyst. It is important to highlight that these results are consistent with DFT findings, in which a theoretical concentration of $25\%$ exhibited higher thermodynamic stability.

**Fig. 16:**
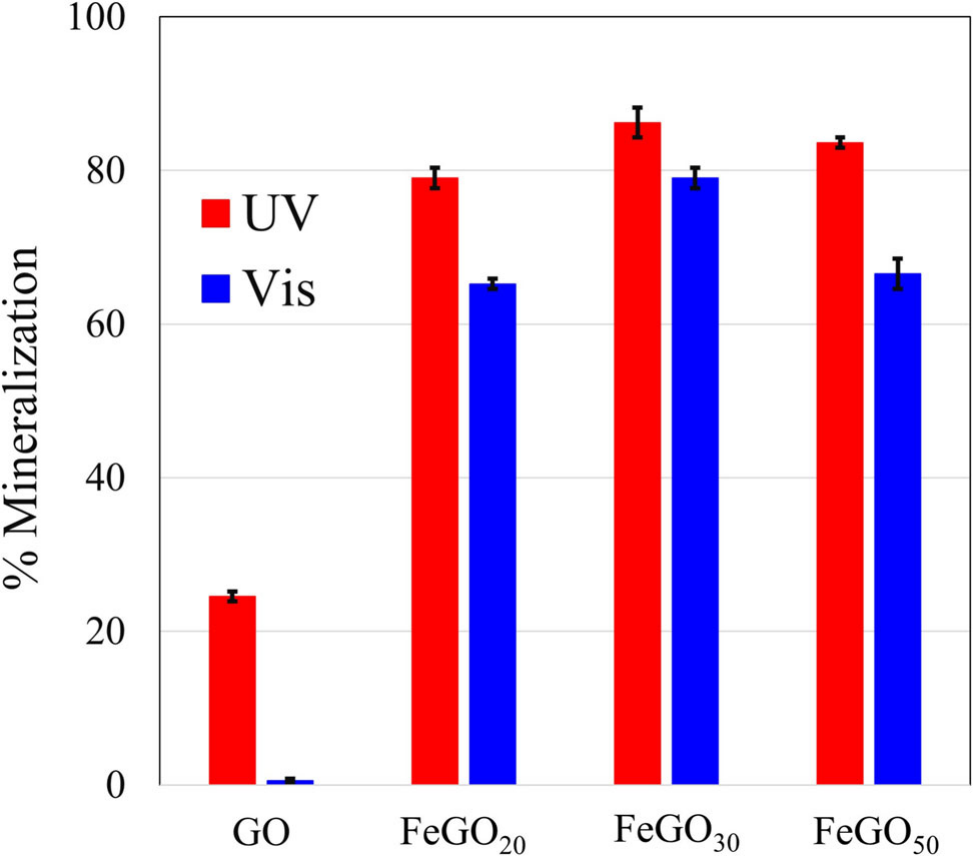
Mineralization process for photocatalytic degradation of acetaminophen with gallium oxide GO and doped with Fe (FeGO_20_, FeGO_30_, and FeGO_50_) treated at ${950\,\mathrm{{ ^{\circ }\text {C}}}}$, under UV and Visible illumination after ${300\,\mathrm{\min }}$

Total organic carbon (TOC) measurements confirmed that Ac mineralizes under the explored photodegradation conditions. We used the Hach direct method for low-range TOC characterization. The results with gallium oxide GO and doped with Fe (FeGO_20_, FeGO_30_, and FeGO_50_) are presented in bar plots in Fig. 16. Experiments were conducted at pH 5 and under UVA and Visible illumination for ${300\,\mathrm{\min }}$. For FeGO_x_ photocatalysts under UVA illumination, $79-86$% of mineralization is reached, while under visible light, the percentages vary between 65 and 80%. GO did not show mineralization under visible illumination, and $\approx 25$% under UVA light. It is important to note that FeGO_30_ showed the highest level of mineralization under both types of illumination. This observation aligns with theoretical computations that suggested the higher stability of this material, which is directly linked to improved optical properties among the studied doping concentrations.

For FeGO_x_, the complete TOC removal was not achieved in 300 min of reaction, neither with UV nor visible illumination, due to the presence of aromatic and aliphatic intermediates formed during the degradation process. Other authors have reported similar mineralization results. Aguilar et al. (2011) reported a photocatalytic degradation of acetaminophen with TiO$_2$ DP25 (2 g L^–1^), achieving 99.9% degradation and $\approx 85\%$ TOC removal after 300 min. Ling et al. (2019) reported TOC removal of 80% at the end of acetaminophen degradation using Ag-g-C$_3$N$_4$ photocatalyst.

**Fig. 17:**
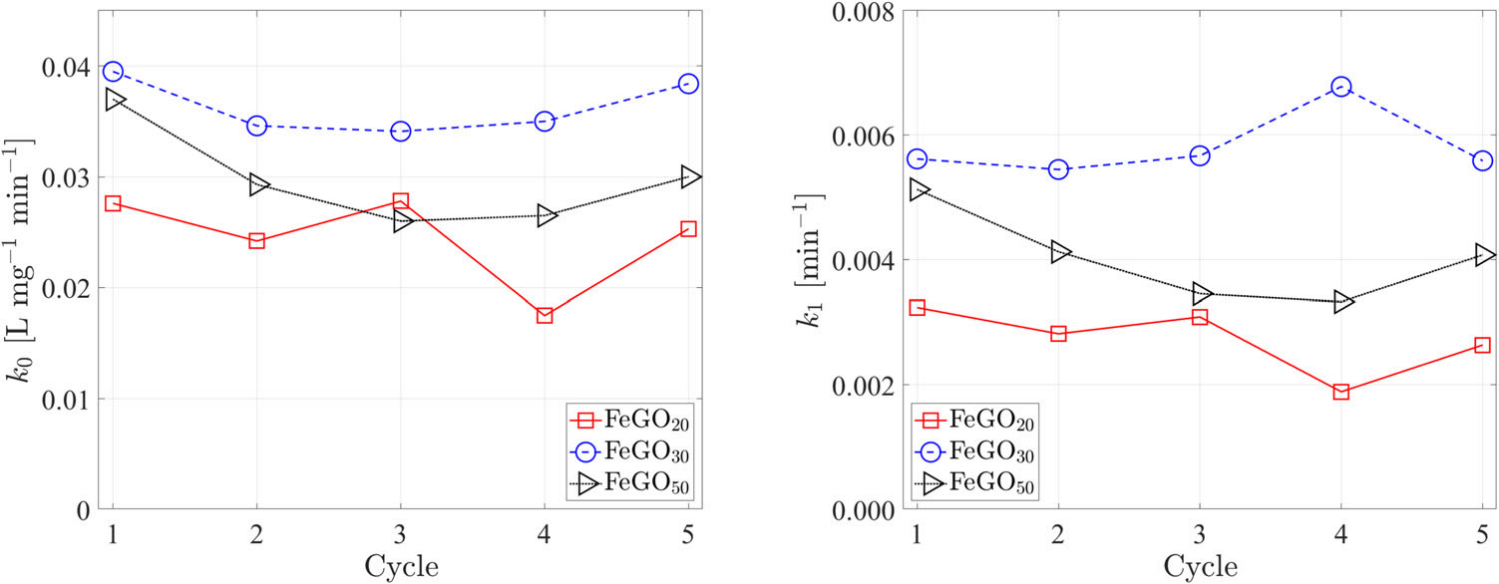
Pseudo-zero ($k_0$ left) and pseudo-first-order ($k_1$ right) kinetic constants for FeGO_20_, FeGO_30_, and FeGO_50_ photocatalysts in five cycles (${300\,\mathrm{\min }}$ each), under visible illumination

A 9-h experiment was carried out at pH 5, using FeGO_30_ at a concentration of 0.5 g L^–1^ and 12 mg L^–1^ of Ac to assess the time required to reach its total mineralization. The degradation process was followed by UV–Vis (see Fig. S12 in SM) and by FT-IR (see Fig. S13 in SM) spectroscopy, while mineralization was evaluated by TOC analysis (see Fig. S14 in SM). Figure S12 (in SM) shows the temporal evolution of the Ac group degradation. As observed, the photocatalytic reaction proceeds to the total degradation of the Ac. Figure S13 (in SM) shows the absorption bands of the Ac functional groups during the photodegradation process obtained by FT-IR. These results reveal the formation of intermediary compounds identified by Aguilar et al. (2022), including N-acetyl-*p*-benzoquinone imine, hydroxylated benzene (*p*-di-hydroxy-benzene, *o*-di-hydroxy-benzene and tri-hydroxy-benzene), 1,4-benzoquinone, and aliphatic compounds to mineralization. The process of mineralization reached 97% in 7 h and 99% after 9 h, as shown in Fig. S14 in the SM.

Catalysts used in practical applications need to be stable under illumination and able to be reused (cycled). The stability study was performed for five cycles with a reaction time of ${300\,\mathrm{\min }}$ each. The Ac degradation profiles for FeGO_x_ photocatalysts for the five cycles under UVA and visible illumination are shown in Figs. S15 and S16 in the SM, respectively. The experiments were carried out at pH 5, 0.5 g L^–1^ of catalyst concentration, and 12 mg L^–1^ of Ac concentration. At the end of the photocatalytic reaction, the photocatalyst was recovered, dried at room temperature, and used for the next experiment. This procedure was repeated for each cycle. The results show that all materials maintained their photocatalytic activity consistently when exposed to UVA and visible light. Additionally, the degradation profiles exhibited similar behavior in all cases. These results show the photocatalytic stability of the tested FeGOx, which are consistent with those reported for other materials such as Cu_2_O/WO_3_/TiO_2_ (Chau et al. 2022, Cu_x_GO (Orozco et al. 2024), immobilized TiO_2_ P25 (Yahiaoui et al. 2024), Zn-Ag co-doped barium hexaferrite-CNTs composite (Irshad et al. 2024), and Ag/ZnO (Mohsentabar et al. 2023).

The values of the kinetic constants, $k_0$ and $k_{1}$, for the five continuous cycles under visible illumination are shown in Fig. 17. The corresponding values are reported in Table S4 in SM. It can be observed that FeGO_30_ exhibited higher values among the three photocatalysts with almost similar values through the five cycles. In turn, for FeGO_20_ and FeGO_50_, the degradation rate presents a slight diminishment of the corresponding values through cycling. Under UVA illumination (see Fig. S15 and Table S4 in SM), all materials exhibit similar kinetic constant values with slightly higher values than in the case of visible illumination, with FeGO_20_ being the photocatalyst that improves the most concerning their performance under visible light (see Fig. S16 and Table S4 in SM). The leached Fe from FeGO_20_, FeGO_30_, and FeGO_50_ photocatalysts in cycles (see Table S5 in SM) after 300 min, are $0.22-0.57$, $0.17-0.32$, and $0.11-0.17$%, respectively. This indicates that all materials are stable under the explored conditions, with FeGO_30_ outperforming the other two materials through the five experiments, which is consistent with DFT findings.

**Fig. 18:**
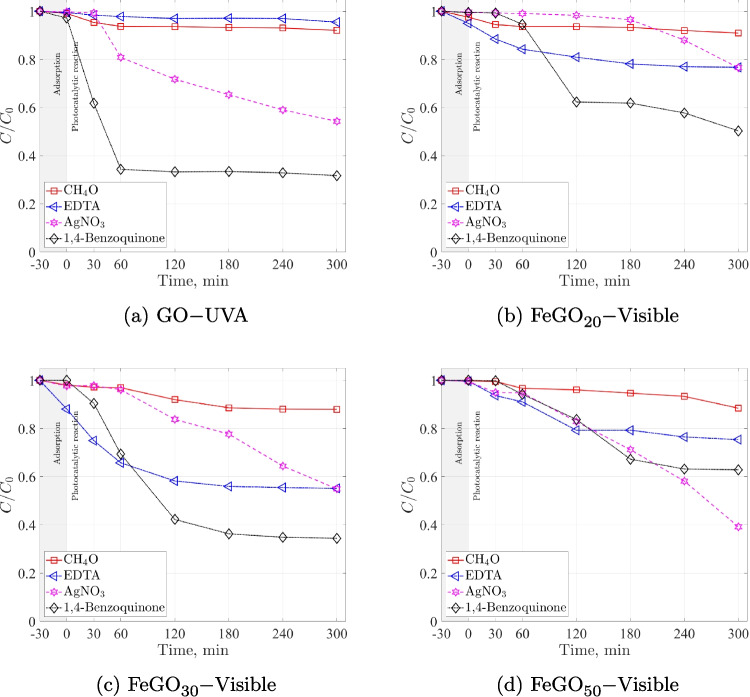
Ac degradation profiles for all photocatalysts using different scavengers

Several experiments were conducted using electron, proton, superoxide, and hydroxyl radical scavengers to elucidate the reaction mechanism. For this purpose, the Ac degradation process was carried out with GO and FeGO_x_ under UVA and visible illumination, respectively. The influence of the explored scavenger on the Ac degradation process with GO, FeGO_20_, FeGO_30_, and FeGO_50_ is shown in Fig. 18.

In the case of GO, the introduction of additional levels in the forbidden band gap can activate the material (Trenczek-Zajac et al. 2022). This activation allows photoexcited electrons to migrate to the CB, leaving behind holes in the VB that can participate in redox reactions. The electrons in the CB of GO cannot reduce oxygen to superoxide radicals since its CB is below the reduction potential of O$_{2}$/O_2_^–•^. However, holes left in the VB can generate HO^•^, as the VB is below the water oxidation potential H$_{2}$O/HO^•^ (see Fig. 12b). In turn, when $e^{-}$ scavengers, such as AgNO$_3$ and 1,4-benzoquinone, are used, a 46 and 68% Ac degradation is achieved (see Fig. 18a), respectively. When $h^{+}$ and HO^•^ scavengers (EDTA-2Na and methanol, respectively) are used, no decomposition of Ac occurs. Hence, Ac degradation with GO primarily occurs through oxidation reactions involving $h^{+}$ and HO^•^.

For FeGO_30_ and FeGO_50_, the photoexcited electrons move to the CB and can reduce oxygen to superoxide radicals since the CB is above the reduction potential of O$_{2}$/O_2_^–•^ (see Fig. 12b). For FeGO_20_, the CB is near the reduction potential of oxygen (see Fig. 12b), and thus, electrons could reduce oxygen to superoxide radicals (Trenczek-Zajac et al. 2022). For FeGO_50_, no HO^•^ are produced because the VB is above the water oxidation potential H$_{2}$O/HO^•^ (see Fig. 12b). HO^•^ radicals are generated with FeGO_20_ and FeGO_30_. When using the O_2_^–•^ scavenger (1,4-benzoquinone), the three FeGO_x_ photocatalysts led to Ac degradation (50, 66 and $33\%$ for FeGO_20_, FeGO_30_ and FeGO_50_, respectively), as shown in Fig. 18. While HO^•^ scavenger (methanol), the three FeGO_x_ photocatalysts did not exhibit a significant Ac degradation. However, when $h^{+}$ and $e^{-}$ scavengers are employed (EDTA-2Na and AgNO$_3$), Ac degradation is observed in all cases. This suggests that degradation can occur via oxidation by HO^•^ radicals for FeGO_20_ and FeGO_30_. Whereas for FeGO_50_, the reaction mechanism mainly occurs via hole oxidation.

Thus, the reaction mechanism could occur by the following: *(i)* attack of HO^•^ radicals on the aromatic ring to form the bis-, tris-, and tetra-hydroxylated intermediate followed by the aromatic ring cleavage that leads to the formation of aliphatic intermediates, or *(ii)* oxidation through holes of the aromatic ring to form hydroquinone and acetamide (Murguía et al. 2025).

## Conclusions

In this work, gallium oxides doped with different atomic ratios of Ga-Fe (100:0, 80:20, 70:30, and 50:50) were synthesized, characterized, and evaluated in the degradation of an emergent pollutant. The study considers theoretical modeling through the density functional theory. The photocatalysts were characterized by different techniques to investigate and corroborate the effect of iron on the structural, optical, and morphological properties. The results showed that the Fe content influences the properties of gallium oxides. The main findings of this work are as follows:

- Using commercial low-power illumination sources (13 W) led to high degradation percentages (up to $\sim 88\%$) achieved at pH 5 under both visible and UVA light sources. When UVA light is used, an 80% degradation is obtained after 180 min. While under visible light, a degradation percentage between $80-88\%$ is reached after 5 h.
- The band gap of gallium oxide is $\sim 4.41$ eV (UV light absorption). After doping with iron, the band gap decreases to 3.21, 3.18, and 2.78 eV for FeGO_20_, FeGO_30_, and FeGO_50_, respectively. The shift occurs via the formation of Fe_2_O_3_ in the photocatalysts.
- The obtained pseudo-first-order kinetic constants range from 0.00324 to 0.00562 min$^{-1}$ under visible illumination. A slight improvement is observed when using UVA light, 0.00653 to 0.00763 min$^{-1}$. These results are consistent with those reported in the literature under similar conditions (illumination power and working volumes).
- These values exhibited a slight variation through 5 cycles.
- For FeGO_X_ photocatalysts under UVA illumination, $79-86\%$ of mineralization is reached after 300 min, while under visible light, the percentages vary between 65 and 80%.
- For the best-performing material and conditions (FeGO_30_ at pH 5, 0.5 g L$^{-1}$ and 12 mg L$^{-}1$), the mineralization reached $97\%$ in 7 h and $99\%$ after 9 h.
- Among the three materials, the experimental results demonstrate that the best-performing material is FeGO_30_ (higher degradation rates, mineralization, and lower leaching percentage) under visible illuminating sources. This is consistent with DFT findings, in which a theoretical concentration of $25\%$ exhibited higher thermodynamic stability.
- Theoretical modeling using DFT corroborated the experimental findings, providing a deeper understanding of the band structure and photocatalytic behavior of the Fe-doped gallium oxide materials.

Finally, it is important to highlight that all Fe-doped photocatalysts showed photocatalytic activity in the visible region, with similar performances under UVA light, making them suitable for use under solar radiation. Although these results have potential for sustainable water remediation, it is necessary to consider several challenges: (i) to scale up the process, (ii) to investigate its application in degrading other emerging pollutants in which the presence of interfering or inhibitory substances might play a crucial role, or even (iii) to treat pollutant mixtures or real wastewater. This requires designing appropriate photocatalytic reactors and immobilizing the photocatalysts. Moreover, the ferromagnetic behavior identified by DFT must be further investigated since it could extend the application of iron-doped gallium oxides. Also, incorporating substitutional dopants affects the pristine environment of $\beta -$Ga_2_O_3_, which may indicate vacancies of O, Ga, and phase segregation requiring more extensive computational methods that address each variant. This is an ongoing work.

## Supplementary information

The data supporting this article have been included as part of the Electronic Supplementary Material (SM).

## Supplementary Information

Below is the link to the electronic supplementary material.

Supplementary file 1 (pdf 11805 KB)

## Data Availability

The datasets used and analyzed during the current study are available from the corresponding author upon reasonable request.
