# Supplementary material for: Simulation and experimentation of iron-doped liquid metal-based gallium oxide photocatalysts for environmental applications harnessing solar energy
Source: Environ Sci Pollut Res Int. 2025 May 8;32(21):12913–44. doi: 10.1007/s11356-025-36436-x (PMC12119720; doi:10.1007/s11356-025-36436-x)
Supplement: Supplementary file 1 — (pdf 11805 KB) [file 11356_2025_36436_MOESM1_ESM.pdf]

Supplementary material

Simulation and Experimentation of Iron-Doped Liquid Metal-Based Gallium Oxide Photocatalysts for Environmental Applications Harnessing Solar Energy

S. Orozco, E. Martínez-Aguilar, C. Belver, J. Bedia and M. Rivero

2025

A Density Functional Theory (DFT)

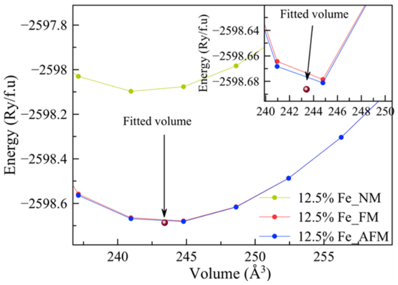

(a)

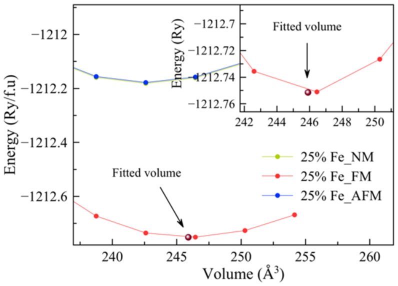

(b)

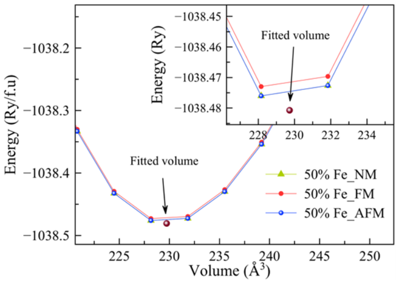

(c)

Figure S1: Equation of states of the possible magnetic, ferromagnetic, antiferromagnetic, and non-magnetic states for gallium doped with Fe at a) 12.5%, b) 25%, and c) 50%.

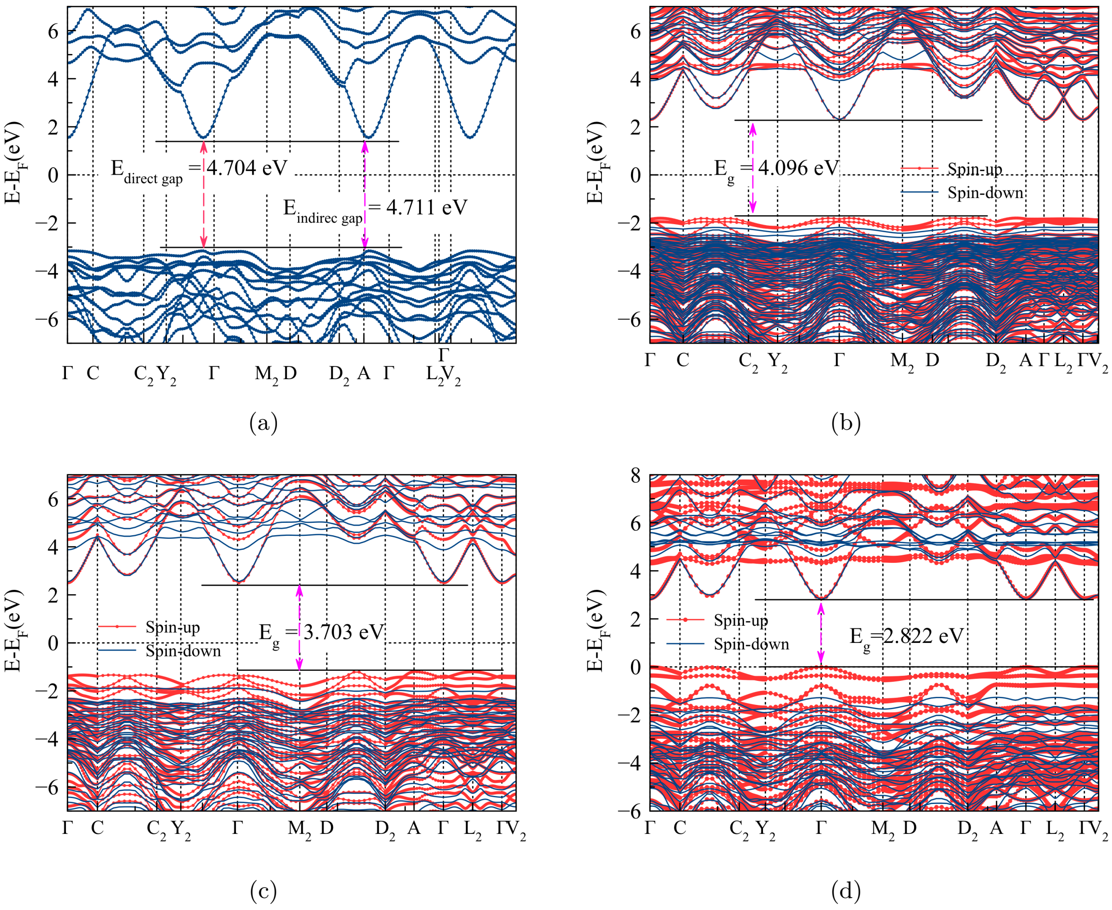

Figure S2: Band structure for Fe-doped gallium at a) 0% b) 12.5%, c) 25%, and d) 50%.

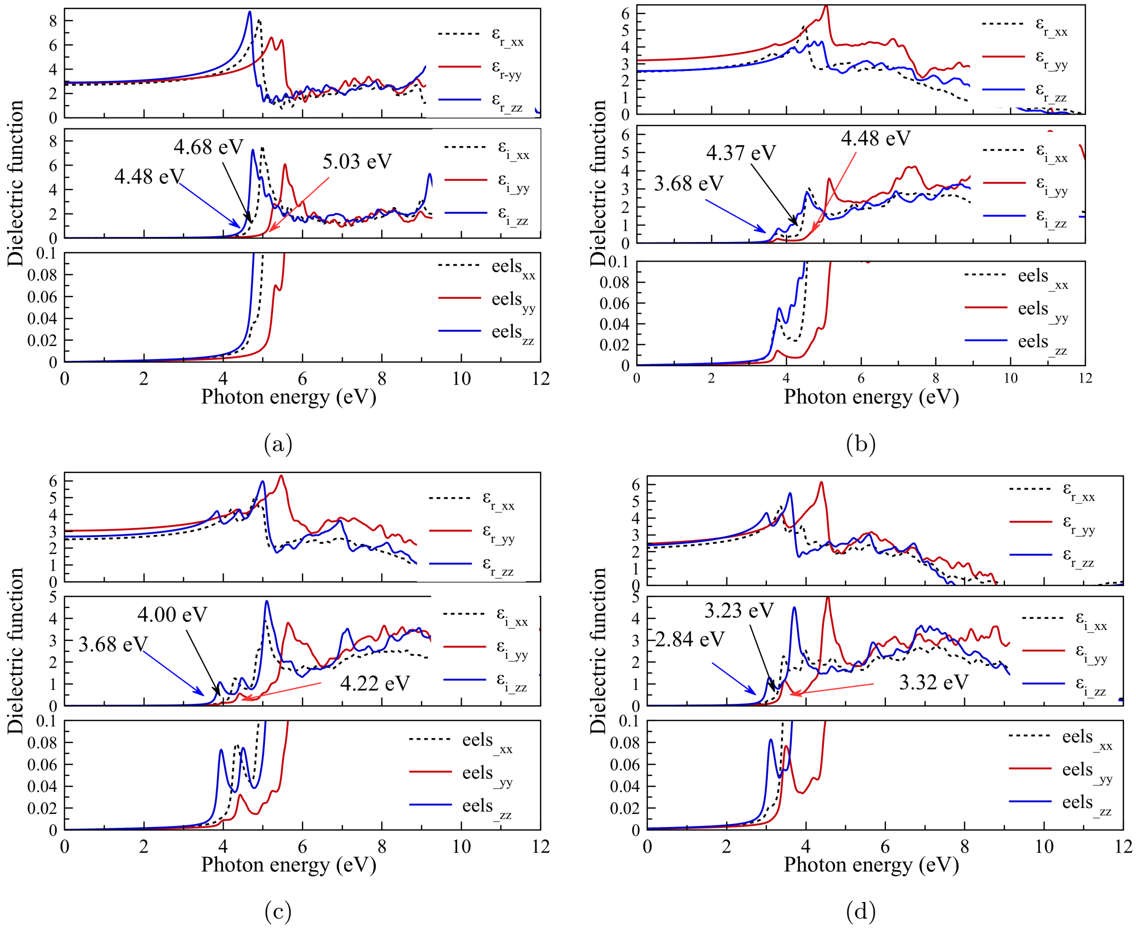

Figure S3: Decomposition of the dielectric function in the different crystallographic axes for Fe-doped gallium at a) 0% b) 12.5%, c) 25%, and d) 50%.

Table S1: Bader charge as a function of Fe doping in β-Ga_2O_3.

**0% Fe**		**12.5% Fe**		**25% Fe**		**50% Fe**	
**Atom**	**Charge (e)**	**Atom**	**Charge (e)**	**Atom**	**Charge (e)**	**Atom**	**Charge (e)**
Ga	2.080403	Ga	2.077993	Ga	1.867044	Ga	2.087938
Ga	2.080358	Ga	2.076191	Ga	1.875313	Ga	2.087912
Ga	2.076251	Ga	2.088124	Ga	1.883847	Ga	2.169174
Ga	2.076251	Ga	2.070089	Ga	1.926438	Ga	2.169174
Ga	2.169494	Ga	2.079154	Ga	1.947259	Fe	2.904839
Ga	2.169494	Ga	2.077132	Ga	1.931287	Fe	2.904826
Ga	2.176796	Ga	2.084136	Fe	2.564957	Fe	2.98695
Ga	2.176796	Ga	2.165088	Fe	2.651255	Fe	2.98695
O	-1.422021	Ga	2.156953	O	-1.282037	O	-1.433445
O	-1.422021	Ga	2.1602	O	-1.272775	O	-1.433445
O	-1.423174	Ga	2.157344	O	-1.27185	O	-1.574532
O	-1.423174	Ga	2.161543	O	-1.363014	O	-1.57467
O	-1.432662	Ga	2.154737	O	-1.295822	O	-1.481178
O	-1.432662	Ga	2.165789	O	-1.278205	O	-1.481178
O	-1.429893	Fe	2.918053	O	-1.274683	O	-1.576302
O	-1.429893	Fe	2.973175	O	-1.366302	O	-1.576302
O	-1.393298	O	-1.416677	O	-1.279362	O	-1.418128
O	-1.393	O	-1.408897	O	-1.268255	O	-1.41801
O	-1.401785	O	-1.408282	O	-1.317704	O	-1.566734
O	-1.401785	O	-1.582	O	-1.277825	O	-1.564929
		O	-1.425038				
		O	-1.410656				
		O	-1.409298				
		O	-1.412849				
		O	-1.484568				
		O	-1.431494				
		O	-1.444491				
		O	-1.508736				
		O	-1.433046				
		O	-1.429074				
		O	-1.437123				
		O	-1.509547				
		O	-1.387636				
		O	-1.388956				
		O	-1.38882				
		O	-1.429532				
		O	-1.390391				
		O	-1.39614				
		O	-1.535519				
		O	-1.396222				

B Characterization

DRX

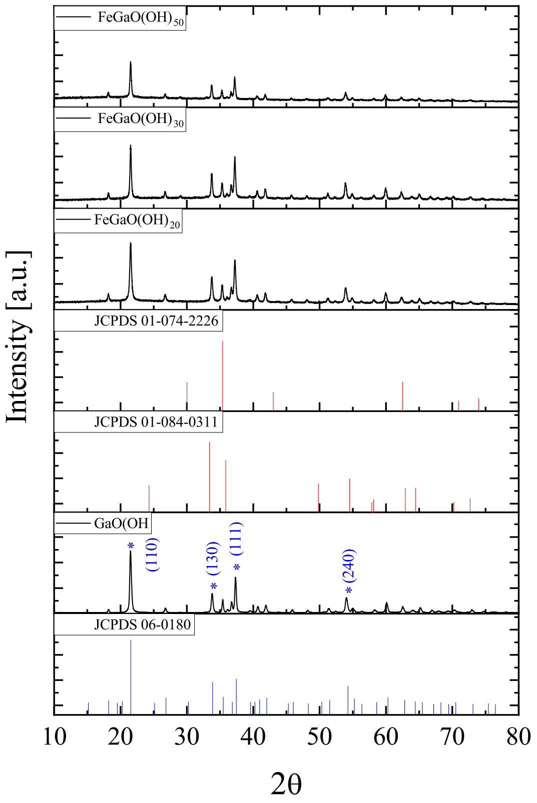

Figure S4: XRD patterns of GaO(OH), and gallium oxi-hidroxide doped with Fe ($FeGaO(OH)_{20}$, $FeGaO(OH)_{30}$ and $FeGaO(OH)_{50}$).

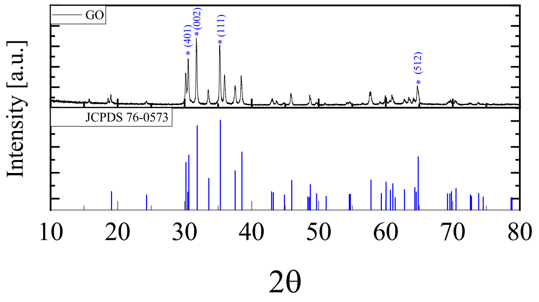

Figure S5: XRD patterns of GO.

SEM

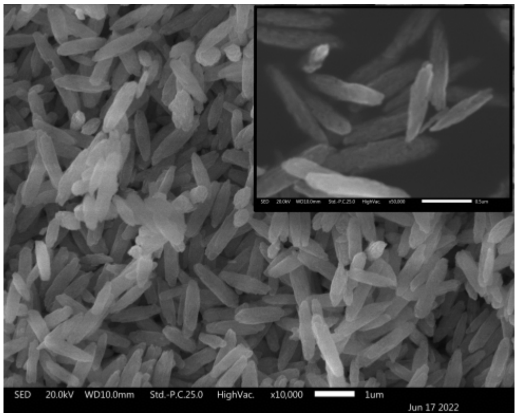

(a) GO & zoom

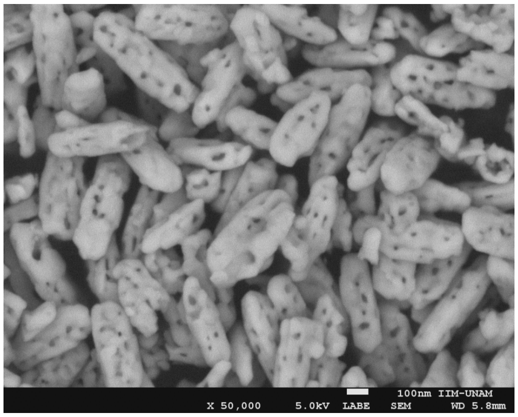

(b) $FeGO_{20}$

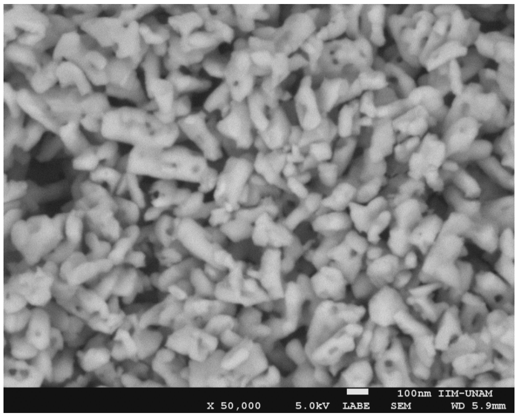

(c) $FeGO_{30}$

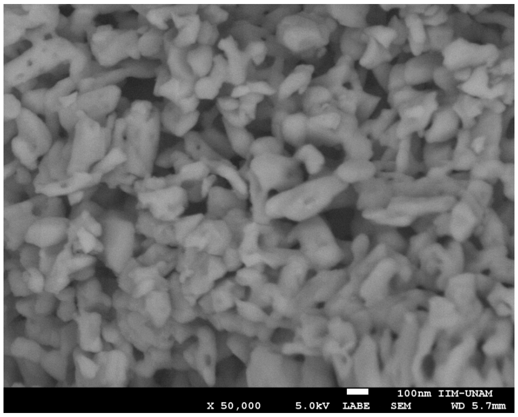

(d) $FeGO_{50}$

Figure S6: SEM for (a) GO, (b) Fe 20%, (c) 30%, and (d) 50% Fe doping.

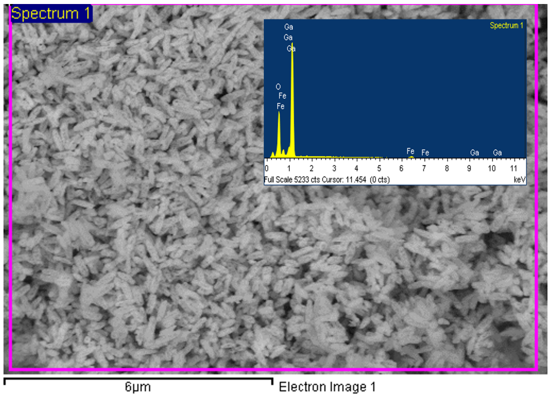

(a) MGFe20

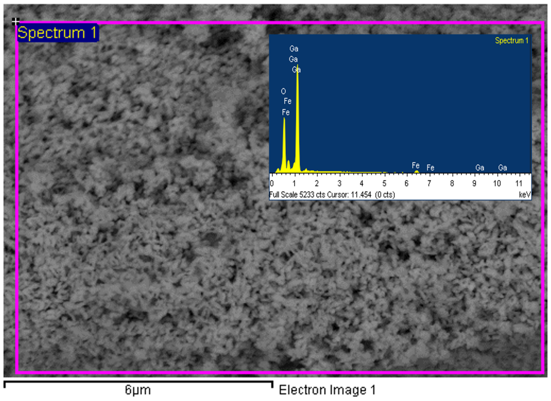

(b) MGFe30

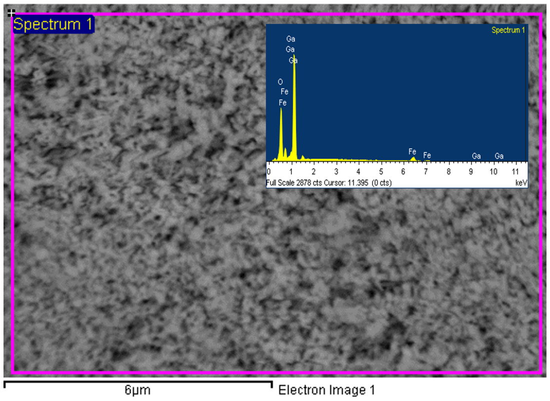

(c) MGFe50

Figure S7: SEM-EDS Spectra for (a) 20%, (b) 30%, and (c) 50% Fe doping.

Table S2: Composition by SEM-EDS.

Photocatalyst	Element	Atomic [%]
$FeGO_{20}$	O	54.56 ± 0.76
	Ga	34.67 ± 0.58
	Fe	10.76 ± 0.41
$FeGO_{30}$	O	54.50 ± 0.26
	Ga	30.69 ± 1.22
	Fe	14.80 ± 1.01
$FeGO_{50}$	O	53.95 ± 0.43
	Ga	30.02 ± 0.29
	Fe	16.02 ± 0.29

TEM

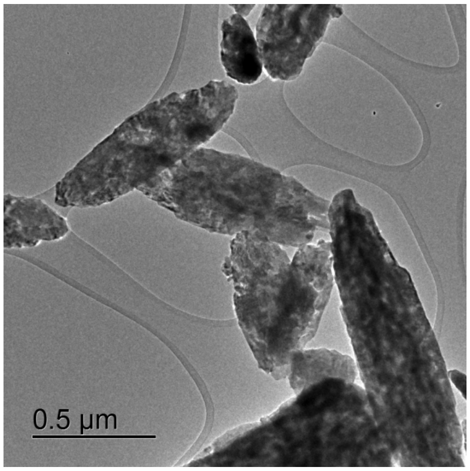

(a) GO

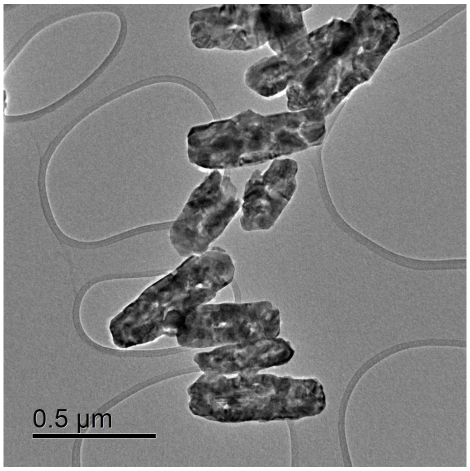

(b) $FeGO_{20}$

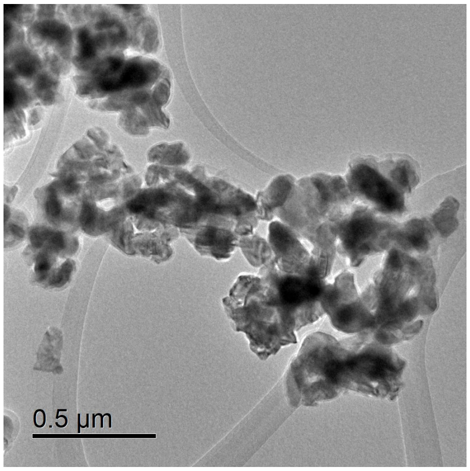

(c) $FeGO_{30}$

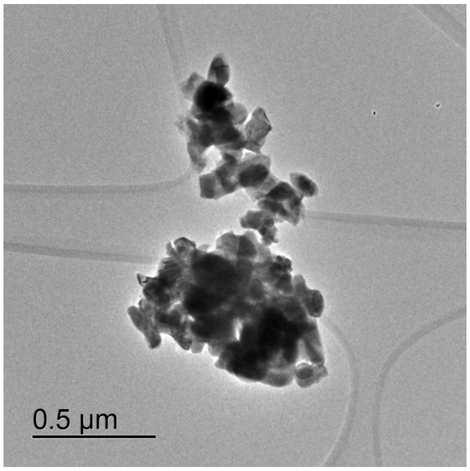

(d) $FeGO_{50}$

Figure S8: Low magnification TEM images of a) β-Ga_2O_3 nanorods, b) nanorods, 20% Fe, c) agglomerate of β-Ga_2O_3 particles at 30% Fe and d) agglomerate of particles β-Ga_2O_3 at 50% Fe.

C Acetaminophen degradation

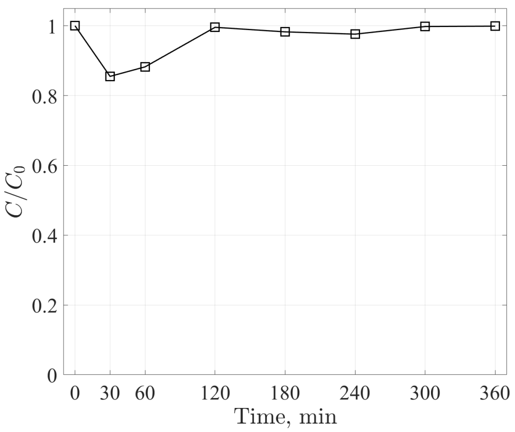

Figure S9: Adsorption of acetaminophen with gallium oxide (Ga_2O_3) for pH 5, 0.5 g L^{-1} of catalyst, and 12 mg L^{-1} of Ac, in absence of illumination.

Table S3: Pseudo zero- and first-order kinetic constants, k_0 and k_1 for GO photocatalyst, under different experimental conditions.

	Value	k_0, L min^{-1} mg^{-1} (R^2)	k_1, min^{-1} (R^2)
pH	3	0.0180 ± 0.0018 (0.9916 ± 0.0024)	0.001815 ± 0.00015 (0.9948 ± 0.0003)
	5	0.0332 ± 0.0017 (0.9846 ± 0.0019)	0.004867 ± 0.00033 (0.9877 ± 0.0016)
	7	0.0179 ± 0.0003 (0.9943 ± 0.0005)	0.001772 ± 0.00003 (0.9945 ± 0.0023)
	9	0.0018 ± 0.0009 (0.8454 ± 0.0650)	0.000153 ± 0.00008 (0.8448 ± 0.0656)
C_{GO}	g L^{-1}	k_0, L min^{-1} mg^{-1} (R^2)	k_1, min^{-1} (R^2)
	0.5	0.0332 ± 0.0017 (0.9846 ± 0.0019)	0.004867 ± 0.00033 (0.9877 ± 0.0016)
	0.75	0.0255 ± 0.0016 (0.9972 ± 0.0022)	0.002594 ± 0.00019 (0.9884 ± 0.0015)
	1.0	0.0245 ± 0.0018 (0.9967 ± 0.0005)	0.002349 ± 0.00020 (0.9868 ± 0.0015)
C_{Ac}	mg L^{-1}	k_0, L min^{-1} mg^{-1} (R^2)	k_1, min^{-1} (R^2)
	6	0.0187 ± 0.0011 (0.9924 ± 0.0048)	0.003929 ± 0.00025 (0.9727 ± 0.0017)
	12	0.0332 ± 0.0017 (0.9846 ± 0.0019)	0.004867 ± 0.00033 (0.9877 ± 0.0017)
	24	0.0382 ± 0.0033 (0.9935 ± 0.0018)	0.001831 ± 0.00016 (0.9927 ± 0.0005)

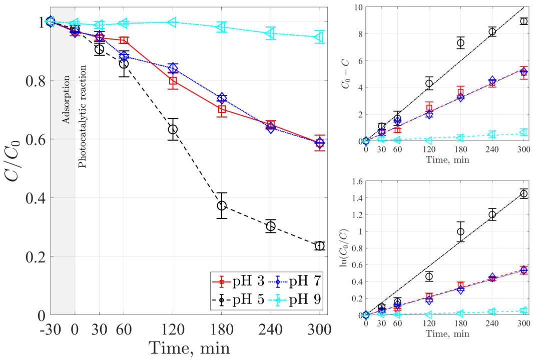

(a) pH

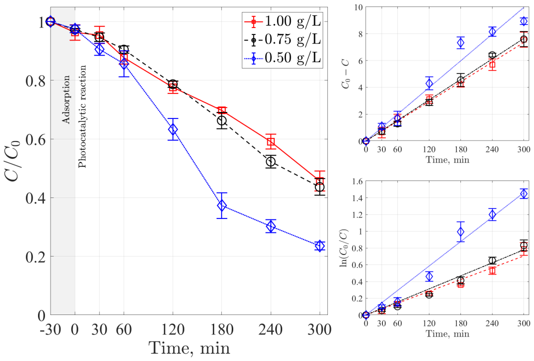

(b) Catalyst

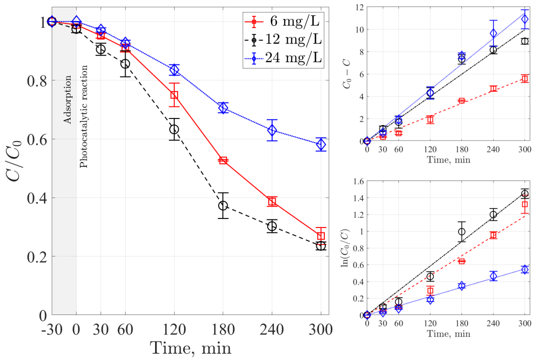

(c) Acetaminophen

Figure S10: (*Left*) Photocatalytic degradation of acetaminophen with gallium oxide (GO) for (a) pH values, (b) 0.5, 0.75 and $1\,\mathrm{g\,L^{-1}}$ of catalyst, and (c) 6, 12 and $24\,\mathrm{mg\,L^{-1}}$ of Ac, under UVA illumination. (*Top-Right*) Pseudo-zero-order fitting. (*Bottom-Right*) Pseudo-first-order fitting.

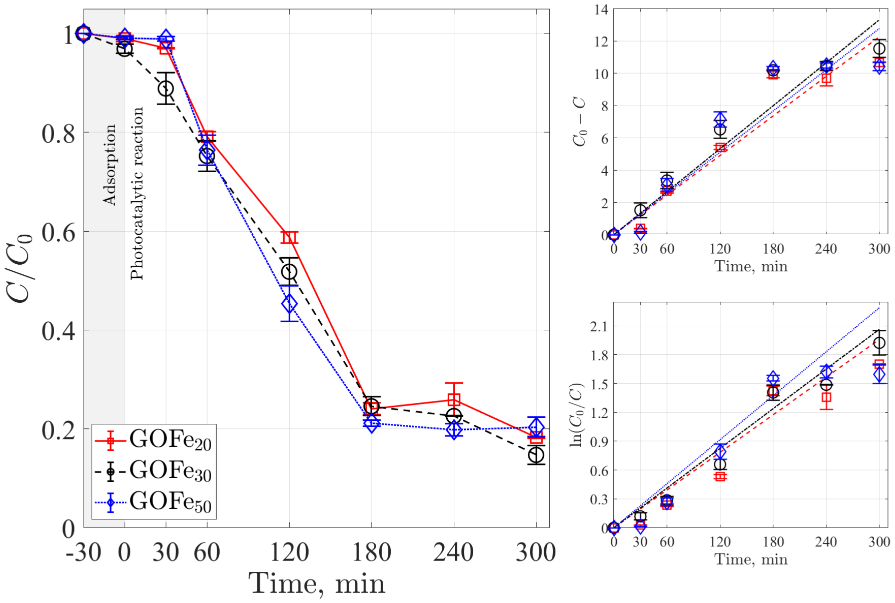

Figure S11: *Left*) Photocatalytic degradation of acetaminophen with $FeGO_x$ at pH 5, $0.5\,g\,L^{-1}$ of photocatalyst and $12\,mg\,L^{-1}$ of Ac under UVA illumination. (*Top-Right*) Pseudo-zero-order fitting. (*Bottom-Right*) Pseudo-first-order fitting.

Table S4: Pseudo zero and first-order kinetic constants, k_0 and k_1 for $FeGO_{20}$, $FeGO_{30}$ and $FeGO_{50}$ photocatalysts in five cycles (300 min each), under UVA and Visible illumination.

Photocatalyst	Cycle	k_0, L mg^{-1} min^{-1} (R^2)		k_1, min^{-1} (R^2)	
		UVA	Vis	UVA	Vis
$FeGO_{20}$	1	0.0415 (0.9690)	0.0276 (0.9424)	0.00653 (0.9249)	0.00324 (0.8842)
	2	0.0425 (0.9850)	0.0242 (0.9012)	0.00954 (0.9609)	0.00282 (0.8312)
	3	0.0451 (0.9583)	0.0278 (0.9871)	0.00902 (0.9857)	0.00309 (0.9674)
	4	0.0464 (0.9574)	0.0175 (0.9813)	0.01008 (0.9831)	0.00189 (0.9617)
	5	0.0459 (0.9756)	0.0253 (0.9917)	0.00706 (0.9980)	0.00264 (0.9953)
$FeGO_{30}$	1	0.0427 (0.9719)	0.0395 (0.9944)	0.00688 (0.9563)	0.00562 (0.9615)
	2	0.0350 (0.9725)	0.0346 (0.9829)	0.00536 (0.9657)	0.00545 (0.9109)
	3	0.0417 (0.9660)	0.0341 (0.9907)	0.00743 (0.9790)	0.00567 (0.9006)
	4	0.0473 (0.9713)	0.0350 (0.9852)	0.00933 (0.9888)	0.00678 (0.8428)
	5	0.0436 (0.9785)	0.0384 (0.9993)	0.00719 (0.9871)	0.00559 (0.9473)
$FeGO_{50}$	1	0.0461 (0.9754)	0.0370 (0.9752)	0.00763 (0.9563)	0.00513 (0.8731)
	2	0.0447 (0.9763)	0.0293 (0.9663)	0.00828 (0.9854)	0.00413 (0.8831)
	3	0.0408 (0.9894)	0.0260 (0.9804)	0.00780 (0.9616)	0.00346 (0.9255)
	4	0.0388 (0.9841)	0.0265 (0.9910)	0.00711 (0.9735)	0.00333 (0.9469)
	5	0.0435 (0.9784)	0.0300 (0.9783)	0.00719 (0.9871)	0.00408 (0.9607)

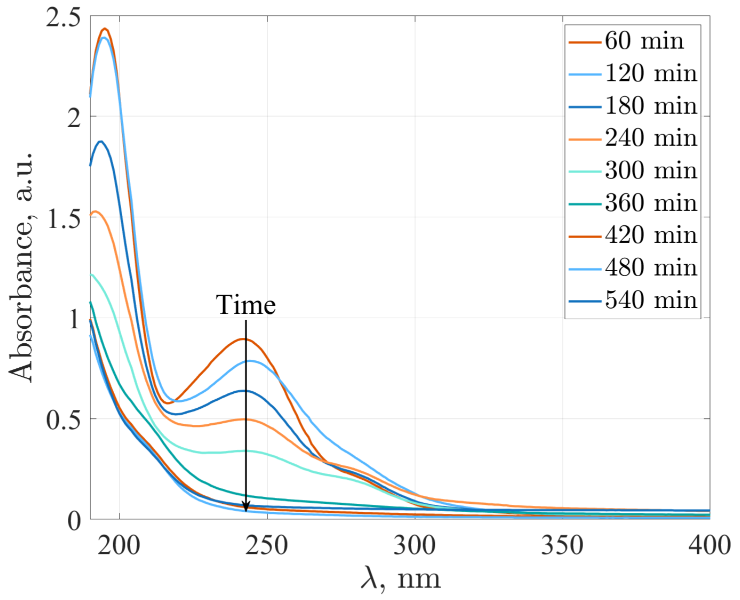

Figure S12: Absorption spectra for the Ac photocatalytic degradation with $FeGO_{30}$ under Visible-UV light. pH 5, $12\,mg\,L^{-1}$ of Ac, and $0.5\,g\,L^{-1}$ of $FeGO_{30}$.

Table S5: Percentage of Fe leached from $FeGO_x$ photocatalysts in cycles.

	**% Fe leached**			
Photocatalysts	Cycle 2	Cycle 3	Cycle 4	Cycle 5
$FeGO_{20}$	0.57	0.30	0.22	0.31
$FeGO_{30}$	0.32	0.22	0.19	0.17
$FeGO_{50}$	0.17	0.13	0.11	0.11

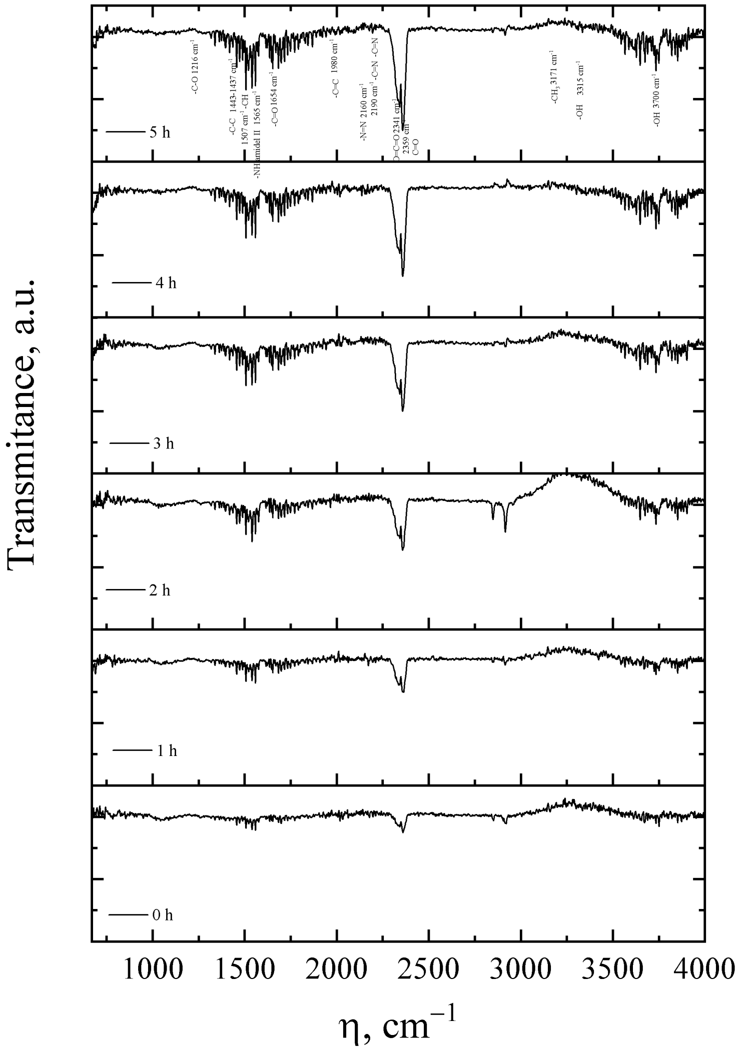

Figure S13: FT-IR spectra for samples of the Ac degradation for $FeGO_{30}$. Experiment was carried out under Visible light at pH 5, $12\,mg\,L^{-1}$ of Ac, and $0.5\,g\,L^{-1}$ of $FeGO_{30}$. The evolution of the photocatalytic degradation process was evaluated by FT-IR spectroscopy. This figure shows the spectra for the six samples. At 0 h, we observe peaks associated with the acetaminophen functional group, as reported by (Aguilar, 2022). Figure shows a change in the intensity of the peaks, during the process, from 1 to 5 h, this is due to the presence of intermediary compounds. The spectra showed the vibrational peaks at 3315 and $3171\,cm^{-1}$, which are assigned to OH and CH_3 stretching, respectively. The peaks at 2341 and $2359\,cm^{-1}$ are associated with $-C{=}O$ and $-O{=}C{=}O$ groups of N-acetyl-p-benzoquinone imine, 1,4-benzoquinone and CO_2. While, the peaks at 2190, 2160, 1980 and $1654\,cm^{-1}$ correspond to $-C{=}N$, $N{=}N$, $-C{=}C$ and $-C{=}O$ stretching, respectively. Finally, vibrational peaks at 1565, 1507 and 1443-1437 cm^{-1} are assigned to -N-H amide II bending, asymmetrical bending in $-C-H$ bend and $C-C$ stretching, respectively (Aguilar, 2022).

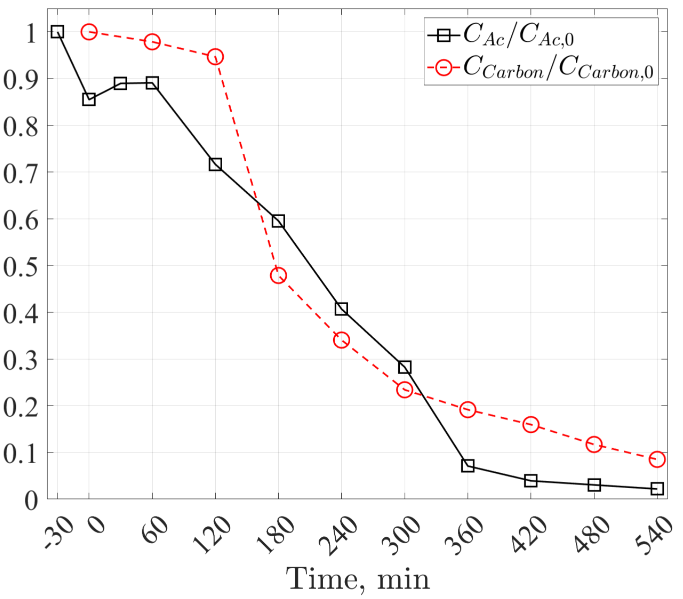

Figure S14: Photocatalytic degradation and mineralization of Ac with $FeGO_{30}$ at pH 5 , $0.5\,g\,L^{-1}$ of catalyst and $12\,mg\,L^{-1}$ of Ac under visible illumination, after 9 hours of reaction time.

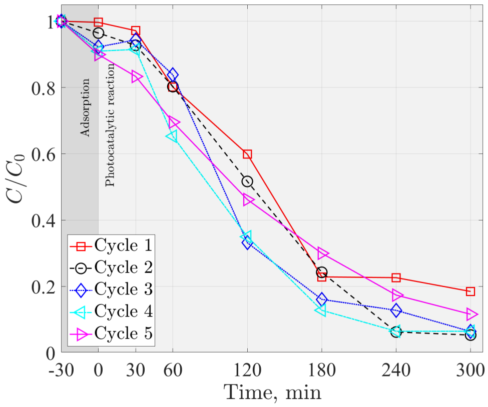

(a) $GaFe_{20}$, UVA

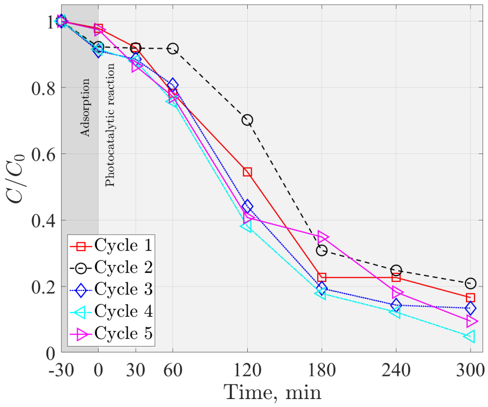

(b) $GaFe_{30}$, UVA

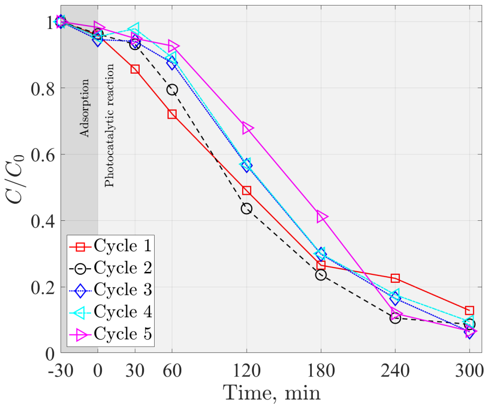

(c) $GaFe_{50}$, UVA

Figure S15: Stability of (a) $FeGO_{20}$, (b) $FeGO_{30}$ and (c) $FeGO_{50}$ photocatalysts, in five cycles (300 min each) of the degradation at experimental conditions: $12\,mg\,L^{-1}$ of acetaminophen, $0.5\,g\,L^{-1}$ of $FeGO_x$, pH 5 and 1.2×10^{-5} of H_2O_2, under UVA illumination.

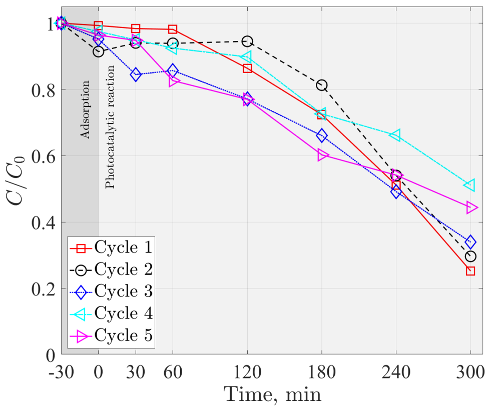

(a) $GaFe_{20}$, Vis

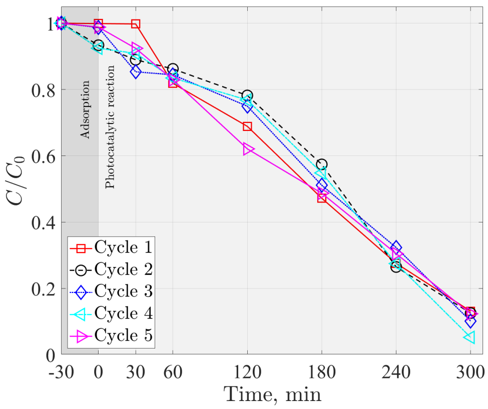

(b) $GaFe_{30}$, Vis

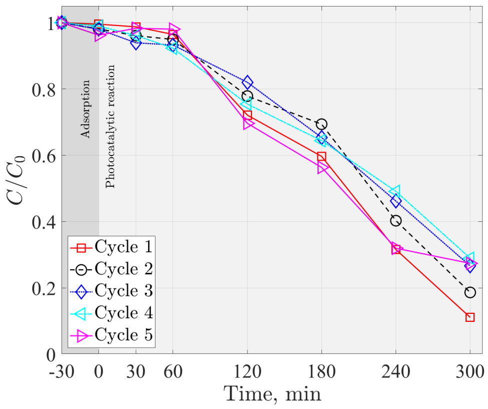

(c) $GaFe_{50}$, Vis

Figure S16: Stability of (a) $FeGO_{20}$, (b) $FeGO_{30}$ and (c) $FeGO_{50}$ photocatalysts, in five cycles (300 min each) of the degradation at experimental conditions: $12\,mg\,L^{-1}$ of Ac, $0.5\,g\,L^{-1}$ of $FeGO_x$, pH 5 and 1.2×10^{-5} of H_2O_2, under Visible illumination.

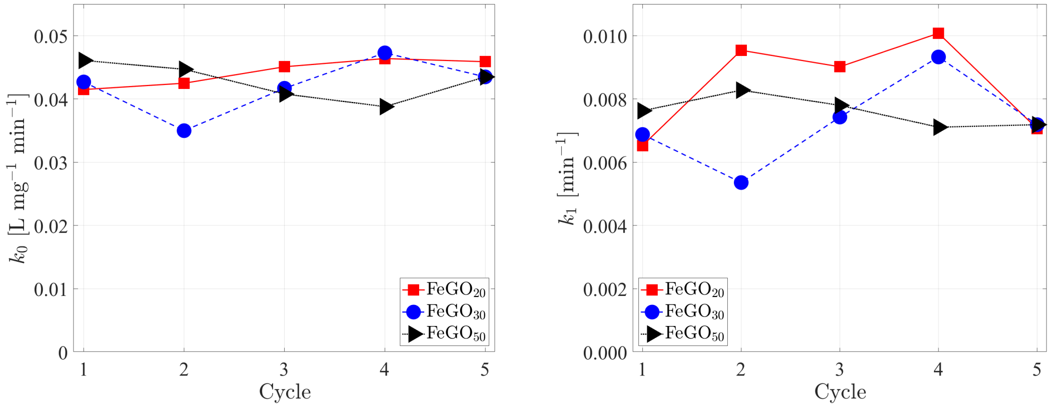

Figure S17: Pseudo zero (k_0 *left*) and first-order (k_1 *right*) kinetic constants for $FeGO_{20}$, $FeGO_{30}$ and $FeGO_{50}$ photocatalysts in five cycles (300 min each), under UVA illumination.
